# A multidisciplinary approach unravels early and persistent effects of X-ray exposure at the onset of prenatal neurogenesis

**DOI:** 10.1186/1866-1955-7-3

**Published:** 2015-01-09

**Authors:** Tine Verreet, Roel Quintens, Debby Van Dam, Mieke Verslegers, Mirella Tanori, Arianna Casciati, Mieke Neefs, Liselotte Leysen, Arlette Michaux, Ann Janssen, Emiliano D’Agostino, Greetje Vande Velde, Sarah Baatout, Lieve Moons, Simonetta Pazzaglia, Anna Saran, Uwe Himmelreich, Peter Paul De Deyn, Mohammed Abderrafi Benotmane

**Affiliations:** Radiobiology Unit, Laboratory of Molecular and Cellular Biology, Institute for Environment, Health and Safety, Belgian Nuclear Research Centre, SCK•CEN, 2400 Mol, Belgium; Laboratory of Neural Circuit Development and Regeneration, Department of Biology, Faculty of Science, University of Leuven, 3000 Leuven, Belgium; Laboratory of Neurochemistry and Behaviour, Institute Born-Bunge, Department of Biomedical Sciences, University of Antwerp, 2610 Wilrijk, Belgium; Laboratory of Radiation Biology and Biomedicine, Agenzia Nazionale per le Nuove Tecnologie, Casaccia Research Centre, l’Energia e lo Sviluppo Economico Sostenibile (ENEA), 00123 Rome, Italy; SB Dosimetry and Calibration, Institute for Environment, Health and Safety, Belgian Nuclear Research Centre, SCK•CEN, 2400 Mol, Belgium; Biomedical NMR Unit, Department of Imaging and Pathology, Faculty of Medicine, University of Leuven, 3000 Leuven, Belgium; Molecular Small Animal Imaging Center (MoSAIC), Faculty of Medicine, University of Leuven, 3000 Leuven, Belgium; Department of Neurology and Alzheimer Research Center, University of Groningen, University Medical Center Groningen, 9713 GZ Groningen, The Netherlands

**Keywords:** Apoptosis, Brain development, Cognitive dysfunction, MRI, Radiation

## Abstract

**Background:**

In humans, *in utero* exposure to ionising radiation results in an increased prevalence of neurological aberrations, such as small head size, mental retardation and decreased IQ levels. Yet, the association between early damaging events and long-term neuronal anomalies remains largely elusive.

**Methods:**

Mice were exposed to different X-ray doses, ranging between 0.0 and 1.0 Gy, at embryonic days (E) 10, 11 or 12 and subjected to behavioural tests at 12 weeks of age. Underlying mechanisms of irradiation at E11 were further unravelled using magnetic resonance imaging (MRI) and spectroscopy, diffusion tensor imaging, gene expression profiling, histology and immunohistochemistry.

**Results:**

Irradiation at the onset of neurogenesis elicited behavioural changes in young adult mice, dependent on the timing of exposure. As locomotor behaviour and hippocampal-dependent spatial learning and memory were most particularly affected after irradiation at E11 with 1.0 Gy, this condition was used for further mechanistic analyses, focusing on the cerebral cortex and hippocampus. A classical p53-mediated apoptotic response was found shortly after exposure. Strikingly, in the neocortex, the majority of apoptotic and microglial cells were residing in the outer layer at 24 h after irradiation, suggesting cell death occurrence in differentiating neurons rather than proliferating cells. Furthermore, total brain volume, cortical thickness and ventricle size were decreased in the irradiated embryos. At 40 weeks of age, MRI showed that the ventricles were enlarged whereas N-acetyl aspartate concentrations and functional anisotropy were reduced in the cortex of the irradiated animals, indicating a decrease in neuronal cell number and persistent neuroinflammation. Finally, in the hippocampus, we revealed a reduction in general neurogenic proliferation and in the amount of Sox2-positive precursors after radiation exposure, although only at a juvenile age.

**Conclusions:**

Our findings provide evidence for a radiation-induced disruption of mouse brain development, resulting in behavioural differences. We propose that alterations in cortical morphology and juvenile hippocampal neurogenesis might both contribute to the observed aberrant behaviour. Furthermore, our results challenge the generally assumed view of a higher radiosensitivity in dividing cells. Overall, this study offers new insights into irradiation-dependent effects in the embryonic brain, of relevance for the neurodevelopmental and radiobiological field.

**Electronic supplementary material:**

The online version of this article (doi:10.1186/1866-1955-7-3) contains supplementary material, which is available to authorized users.

## Background

Central nervous system development is a protracted process comprising a dynamic equilibrium between proliferation, differentiation, migration, synaptogenesis, myelination and cell death [1]. Disturbance of any of these events, both pre- or postnatally, might have serious consequences for adult brain structure and function [2]. Ionising radiation is, amongst other stressors like ethanol, drugs and infectious agents [3], known to cause such a perturbation of brain development, although effects are strongly dependent on the timing and dosage of exposure [4]. Knowledge about the long-term impact of prenatal irradiation has been largely based on epidemiological studies of atomic bomb survivors, in which children *in utero* exposed to radiation had a higher incidence of severe mental retardation and growth impairment [5]. In addition, a decrease in intelligence and school performance and an increased risk for seizures were noted [6]. Since pregnant women can be exposed to radiation during medical examination [7] and/or treatment, particularly during radiotherapy [8], a high concern and the clear need for increased knowledge exists concerning the long-term outcome of the exposed embryo. The risk for a mother to develop cancer during pregnancy is rare (about one in 1,000 to 2,000 pregnancies), but when it occurs, radiotherapy can be considered as treatment during the first and second trimesters. However, the dose to the embryo should be kept as low as reasonably achievable [9]. Successful radiotherapy treatment of pregnant women with breast cancer and Hodgkin’s disease has been reported, with the birth of healthy children [8]. However, it should be noted that so far, these *in utero* exposed patients have been followed up only until childhood. Therefore, the possibility of late side effects, such as subclinical cognitive problems, cannot be excluded.

Epidemiological evidence further suggests that radiation-induced long-term neuronal effects only arise when the exposure occurred between weeks 8 and 15 of pregnancy and to a lesser extent between weeks 16 and 25 [5, 6]. The timing of irradiation thus seems decisive for the manifestation of late effects. The period of weeks 8 to 15 of human gestation is characterised by a fast increase in neuronal cell number, as well as the start of neuronal differentiation and the consequent migration to the cortical plate [10]. Of note, during this developmental period, only a limited migration and final cell positioning takes place. Indeed, by the 20th week of gestation, the cortical plate consists of less than 6 × 10^9^ cells [11], which only accounts for approximately 25% of the adult neocortical cell number [12]. This thus implies that the remaining 75% of neurons still need to migrate to their final destination in the cortex. In the mouse, the radiosensitive period of neurogenesis roughly takes place between embryonic days (E) 12 and E16 [13], whereas the onset of murine neurogenesis occurs at E10 [2, 14], shortly after neural tube closure [15]. In animal models, irradiation at other developmental time points than those corresponding to weeks 8 to 15 in humans have also been studied [16, 17]. In contradiction to epidemiological evidence, they indicate the possibility of neurological deficits outside the most radiosensitive developmental period. For example, in non-human primates, radiation exposure before neurogenesis but after neural tube closure has been shown to result in adult-onset cognitive impairment [18]. Interestingly, *in utero* brain development in rhesus monkeys occurs slow when compared to rodents, allowing irradiation after neural tube closure, but still before the onset of neurogenesis [19]. This specific developmental time window cannot be investigated in mice, as neural tube closure coincides with the start of neurogenesis [20]. Thus, although evidence indicates that irradiation at the onset of neurogenesis may also induce long-term effects, at present this has not yet been studied in a rodent model.

Therefore, as an attempt to study radiosensitivity outside of the currently proposed radiosensitive period in a mouse model, we irradiated animals at the start of neurogenesis, i.e. at E10, E11 or E12 and we performed a detailed investigation to delineate short- and long-term radiation injury to the brain. In a first instance, we assessed radiation effects by subjecting the offspring to a behavioural test battery at the age of 12 weeks. This showed that behavioural changes due to radiation exposure were timing-specific. Irradiation at E10 resulted in an impaired consolidation of reference memory without spatial learning effects, whereas animals exposed at E11 showed both spatial learning and long-term memory deficits. When the irradiation occurred at E12, no changes in learning and memory could be observed. Therefore, we performed a multidisciplinary study in order to unravel early and persistent radiation-induced mechanisms, with a focus on mice irradiated at E11. Hereto, we combined multiple *in vivo* magnetic resonance imaging (MRI) paradigms, as well as gene expression analyses and immunohistochemistry, specifically focusing on the cerebral cortex and hippocampus. With this study, we establish a possible link between early radiation-induced effects and the long-term functional and morphological outcome.

## Methods

### Animals and irradiation

All animal experiments were performed in accordance with the European Communities Council Directive of November 24, 1986 (86/609/EEC) and approved by the local ethical SCK•CEN/VITO (ref. 02–012), University of Antwerp and KU Leuven committees. C57Bl/6 J were purchased from Janvier (Bio Services, Uden, The Netherlands) and housed under standard laboratory conditions (12-h light/dark cycle). Food and water were available *ad libitum*. Female mice were coupled during a 2-h time period in the morning, at the start of the light phase (7.30 h until 9.30 h), in order to ensure synchronous timing of embryonic development. Subsequently, pregnant females were whole-body-irradiated at E10, 11 or 12 (0.1, 0.2, 0.5 or 1.0 Gy) at a dose rate of 0.35 Gy min^-1^ using a Pantak RX tube operating at 250 kV and 15 mA (1-mm Cu-filtered X-rays). The calibration of the X-ray tube was performed using an ionisation chamber. Control mice were sham-irradiated. The offspring was used in further experiments.

### Behavioural tests

At the age of 12 weeks, male animals (irradiated at E10, 11 or 12 with a dose of 0.0, 0.2, 0.5 or 1.0 Gy) were used in behavioural tests. At least eight animals were used in each test (except for the 1.0-Gy irradiated group at E12 in the Morris water maze (MWM) and cage activity (*N* = 4) and for the 0.5-Gy irradiated group at E12 in the rotarod (*N* = 4)).

#### MWM learning and memory

The MWM setting consisted of a circular pool (diameter: 150 cm, height: 30 cm) surrounded by invariable visual extramaze cues. The water was opacified with white non-toxic paint and kept at 25°C. A round acrylic glass platform (diameter: 15 cm) was placed 1 cm below the water surface at a fixed position. The acquisition or training phase comprised eight trial blocks of four daily trials starting from four different positions around the border of the maze in a semi-random order with a 15-min inter-trial interval. In case an animal was unable to reach the platform within 120 s, it was placed on the platform for 15 s before being returned to its home cage. Animals’ trajectories were recorded using a computerised video-tracking system (EthoVision, Noldus, The Netherlands) logging escape latency. Four days after the final acquisition trial block, a probe trial was performed. For this purpose, the platform was removed from the maze and each mouse was allowed to swim freely for 100 s. Spatial acuity was expressed as the percentage of time spent in the four different quadrants of the MWM.

#### Cage activity recording

Ambulatory cage activity was measured in solitary housed animals using standard transparent mouse cages (22.5 × 16.7 × 14 cm, length × width × height) placed between three infrared beams (two perpendicular to and one parallel with the length of the cage). Sensors were positioned 3 cm above the nesting material and detected horizontal activity. Cages were placed in closed cabinets accommodated with electricity-driven ventilation fans to keep optimum temperature and lights to imitate the animals’ 12-h light/dark cycle. The number of beam interruptions in a 23-h period was counted using a microprocessor counter linked to a computer running in-house developed software. Recording started at 16 h and ended at 15 h. Counts were summed over subsequent 30-min periods.

#### Accelerating rotarod

Motor coordination and equilibrium were tested on an accelerating rotarod apparatus (Ugo Basile, Italy). After two adaptation trials of a maximum of 2 min each at a constant speed of 4 rpm, a mouse was placed on the rotating rod for four test trials with an inter-trial interval of 1 min. The time (latency) a mouse could hold itself on the rod was measured up to a maximum of 5 min, during which the rotation speed gradually increased from 4 to 40 rpm.

#### Statistics

Statistical analyses were performed using SigmaStat software (SPSS Inc., Erkrath, Germany). We applied a two-way ANOVA with treatment and quadrant as sources of variation to assess spatial memory in the MWM probe trial. For tests repeated over time (e.g. MWM acquisition), we used a two-way repeated measures (RM) ANOVA. Into more detail, two-way RM ANOVA was used to evaluate the significance of differences between latency scores during acquisition trial blocks in the MWM. Treatment group and trial block were considered as possible sources of variation. In case of a significant *P* value, a *post hoc* Tukey multiple comparison test was performed. For cage activity, we examined general differences in activity patterns using two-way RM ANOVA with treatment and time (i.e. subsequent summed 30-min activity counts) as sources of variation. Rotarod latency was assessed using two-way RM ANOVA with irradiation dose and trial as possible sources of variation. If required, Tukey multiple comparison was applied. Statistical analyses were performed at a significance level of 0.05.

### MR experiments

At 1, 4, 12, 20 and/or 40 weeks after birth, *in utero* irradiated female and male mice (0.0 (*N* = 5) or 1.0 Gy (*N* = 6) at E11) were imaged using *in vivo* MRI. For these MR experiments, anaesthesia was induced in an induction chamber with 3–4% isoflurane in 100% oxygen. In the MR scanner, the anaesthesia was maintained at 1–2% isoflurane in 100% oxygen. Respiration and body temperature were monitored throughout the measurements and maintained at 70–90 min^-1^ and 37°C, respectively. MR images and spectra were acquired using a 9.4-T Biospec small-animal MR scanner (Bruker BioSpin, Ettlingen, Germany) with a horizontal bore of 20 cm and equipped with actively shielded gradients (600 mT m^-1^). We also used a 7.2-cm linearly polarised resonator for transmission and an actively decoupled, circular polarised mouse head surface coil for receiving (both Bruker BioSpin). After acquisition of 2D multi-slice localizer images, 3D T2-weighted MRI, diffusion tensor imaging (DTI) and single-volume MR spectroscopy (MRS) were performed. In order to reduce scanning time, and therefore extended periods of anaesthesia that might affect brain development, DTI measurements were performed only at 12 and 40 weeks after birth and MRS at week 40.

#### 3D T2-weighted MRI

A rapid acquisition with refocused echoes (RARE) protocol was acquired to obtain 3D T2-weighted MR images using the following parameters: echo time (TE) = 12 ms, repetition time (TR) = 1,000 ms, RARE factor = 10, field of view (FOV) = 2.0 × 1.28 × 1.28 cm^3^, matrix = 200 × 128 × 128, isotropic resolution = 100 μm, number of averages = 1. For repeated analysis of the brain structures (motor cortex and ventricles), we identified these structures on every 3D T2-weighted MR image (axial view) based on the brain atlas of Franklin and Paxinos [21]. Confirmation was based on the MRI-based brain atlas provided by the National University of Singapore (http://www.bioeng.nus.edu.sg/cfa/mouse_atlas.html) [22].

The thickness of the motor cortex was manually measured in the axial slice in which lateral ventricles and the dorsal third ventricle meet (illustrated in Additional file 1: Figure S1A). The distance between the corpus callosum (hypointense structure) and the boundary of the brain was measured.

The volume of the ventricles was semi-manually delineated using the region of interest tool of the ParaVision software (Bruker BioSpin). A seeding point was defined in the hyperintense ventricles (Additional file 1: Figure S1B). The automatic delineation of the hyperintense ventricles was manually corrected.

#### DTI

DTI data were acquired using an echo planar imaging (EPI) sequence with spin echo read-out. The following parameters were used: TE = 32 ms, TR = 5,000 ms, *b* values = 0.125 s mm^-2^, number of gradient encoding directions = 21, 20 slices (no gap) of 0.5-mm thickness, FOV = 2.6 × 1.9 cm^-2^, matrix 254 × 192, in-plane resolution = 100 μm, number of averages = 10. Fractional anisotropy (FA) values were determined using the DTI reconstruction tool of the ParaVision 5.1 software (Bruker BioSpin). Regional FA was obtained by placing spherical regions of interest in the respective brain regions using the ParaVision software. Brain regions were identified in the 3D T2-weighted MR images and transferred to the DTI data set that was acquired during the same anaesthesia session. The brain regions studied were the corpus callosum (1), sensory-motor cortex (2), hippocampus (3) and thalamus (4) (Additional file 1: Figure S1C).

#### MRS

**Figure 1:**
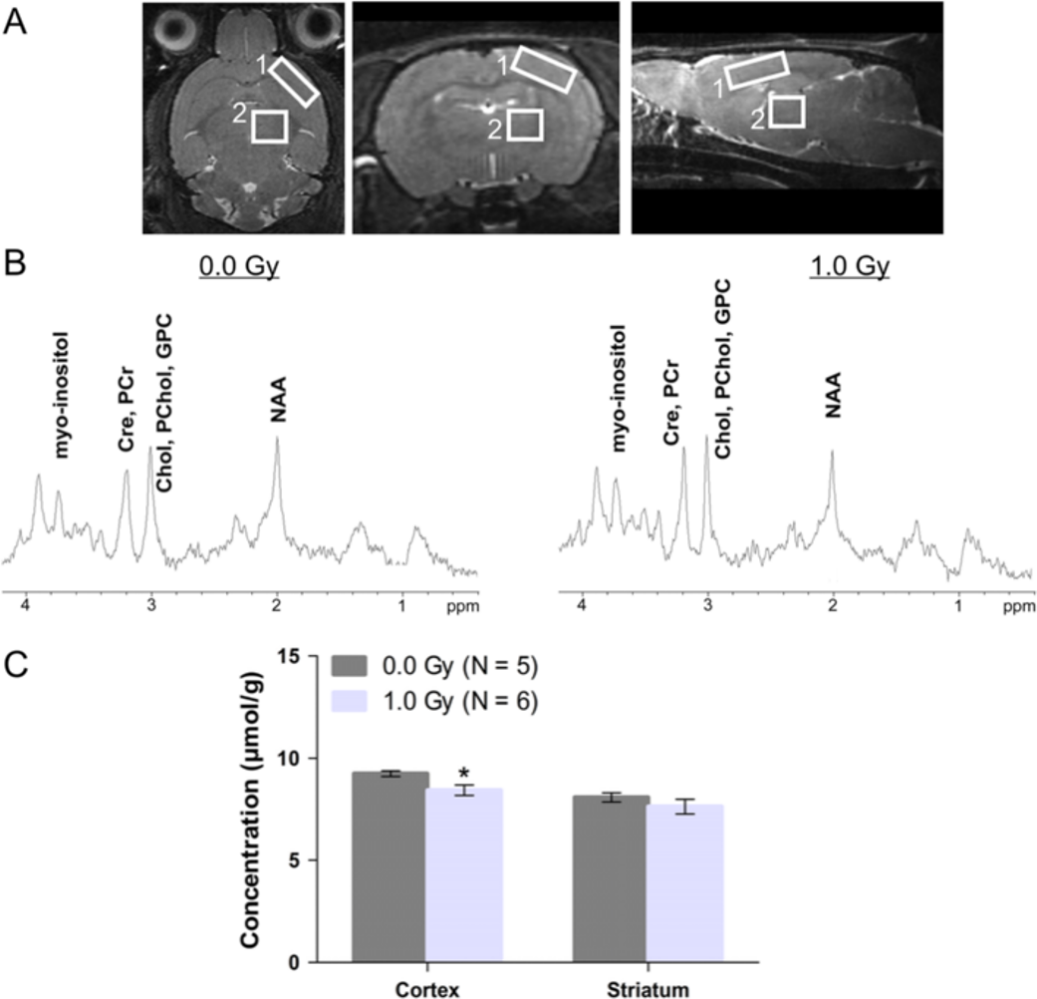
**Neuron loss in the adult cortex of the irradiated animals, indicated by a reduction in NAA. (A)** Localised MR spectra were acquired from volumes of interest, comprising the cortex (1) and a subcortical region (corresponding to the striatum, 2). **(B)** Two representative MR spectra, with indication of the metabolites, acquired from the striatum, are shown. Cre: creatine, PCr: phosphocreatine, Chol: choline, PChol: phosphorylcholine, GPC: glycerophosphorylcholine, NAA: N-acetyl aspartate. **(C)** Quantification of the NAA signal in animals at 40 weeks after birth indicated significantly decreased concentrations in the cortical region, but not in the striatal region, in animals irradiated with 1.0 Gy at E11 when compared to controls. Quantification of all other metabolites is shown in Additional file 3: Table S2. Data are presented as mean ± SEM. *P < 0.05 for comparison with controls. The number of animals used per test is indicated in the graphs (*N*).

Single-voxel 1H MRS was performed as described previously [23, 24]. In brief, the PRESS pulse sequence with implemented pre-delay outer volume suppression, as well as the water suppression method VAPOR, were used. MRS parameters were as follows: TR = 2 s, TE = 20 ms, spectral width = 4 kHz, number of averages = 256 to 400. Volumes of interest (voxels) were placed in the region of the cortex (1.3 × 1.3 × 2.8 mm^3^) (1) and striatum (1.7 × 2.4 × 1.4 mm^3^) (2) (as illustrated in Figure 1A). Spectra were corrected for B0 instability due to Eddy currents and B0 drift using the Bruker built-in routines. Shimming was performed using FASTMAP for initial shimming and subsequently by manual shimming on the selected volumes, which resulted in a final water line width of <25 Hz. MR spectra were further processed using the jMRUI software [23, 24]. This analysis included filtering out the residual water signal, phase correction and baseline correction. Signal quantification was performed using AQSES by fitting a linear combination of metabolite profiles to the experimental data and by modelling of the baseline using splines [25]. The basis set of metabolites included alanine, aspartate, creatine and phosphocreatine, γ-aminobutyrate, glucose, glutamine and glutamate, glycerophosphorylcholine and phosphorylcholine, lactate, myo-inositol, N-acetyl aspartate (NAA), phosphoryl ethanolamine and taurine. The unsuppressed water signal was acquired with identical parameters, except for the acquisition of only one average, a higher receiver gain and TR = 10 s. The unsuppressed water was used as an internal reference for the quantification of metabolites.

#### Statistics

Statistical analyses were performed using GraphPad Prism 5.0 (GraphPad Software, San Diego, CA, USA). Statistical significance was determined using two-way RM ANOVA for 3D T2-weighted MRI. ANOVA pairs of means were compared using the Bonferroni *post hoc* test. FA- and MRS-calculated concentration comparisons were performed using a two-tailed Student’s *t*-test. *P* values <0.05 were considered to be statistically significant.

### Gene expression analysis

In order to identify early molecular mechanisms related to radiation exposure, embryonic brains were dissected and frozen for subsequent RNA extraction at 2 and 24 h after irradiation at E11 (0.0, 0.1, 0.2, 0.5 or 1.0 Gy). For both microarrays and quantitative reverse transcriptase PCR (qRT-PCR), at least three embryos from different litters were used.

#### Microarray preparation and analysis

Total RNA was extracted from flash-frozen embryonic brains using the AllPrep DNA/RNA/Protein Mini Kit (Qiagen, Hilden, Germany) and quality-controlled using the 2100 BioAnalyzer (Agilent, Santa Clara, CA, USA). Only samples with a RNA integrity number >8 were used for hybridisation onto Affymetrix Mouse Gene 1.0 ST arrays (Affymetrix, Santa Clara, CA, USA) as per the manufacturer’s recommendations. CEL files were uploaded to the Partek Genomics Suite (version 6.6). Data normalisation was performed using a customised Robust Multi-chip Analysis algorithm (background correction for entire probe sequence, quantile normalisation, log2 transformation of intensity signals). Microarray data are available in the ArrayExpress database (http://www.ebi.ac.uk/arrayexpress) under accession number E-MTAB-2720.

#### Statistics

To identify radiation-responsive genes, we first performed a filtering step, in which we excluded the 75% genes which showed the lowest variance across the different conditions. Next, we performed a two-way ANOVA with dose and scan date as possible sources of variation to account for differences due to possible batch effects. Significantly differentially expressed genes were identified as those with a *P* value <0.05 after correction for multiple testing according to the method of Benjamini and Hochberg [26].

#### Functional enrichment analysis

For functional enrichment analysis, we used the GOrilla tool [27] with the following settings: Organism: *Mus musculus*, Running mode: Two unranked lists of genes (target list: differentially expressed genes, background list: genes expressed above background in at least 30% of all samples, *P* value threshold: 0.001). The results of this analysis were subsequently reduced using REVIGO [28], with default settings. REVIGO serves to remove redundant Gene Ontology (GO) terms. The version of the GO used was as follows: go_201304-termdb.obo-xml.gz.

#### Chromatin immunoprecipitation enrichment analysis

Chromatin immunoprecipitation (ChIP) enrichment analysis [29] was performed to identify putative transcriptional regulators of differentially expressed genes. It combines published data from ChIP-chip, ChIP-seq, ChIP-PET and DamID experiments in order to rank transcription factors that are most likely responsible for observed gene expression changes based on statistical enrichment analysis. For this analysis, differentially expressed genes were used as input and databases from all species, cell types and ChIP methods were interrogated.

#### qRT-PCR

Complementary DNA was prepared from total RNA using the GoScript™ Reverse Transcriptase kit (Promega, Leiden, The Netherlands) using 1 μl of random hexamer primers and 3.75 mM MgCl_2_ in 20-μl reactions. For quantitative PCR, we used the MESA GREEN kit (Eurogentec, Seraing, Belgium) according to the manufacturer’s instructions. Briefly, duplicate 25-μl reactions were performed using 200 nM of forward and reverse primers. Reactions were run in an Applied Biosystems 7500 Fast Real-Time PCR instrument with an initial hold cycle of 5 min at 95°C followed by 40 cycles of denaturation for 3 s at 95°C and primer annealing/elongation for 45 s at 60°C. Afterwards, a melting curve was performed to check for additional PCR products or primer dimers. For each of the tested primer pairs, we first assessed the reaction efficiencies by running standard curves from cDNA samples in which the respective genes were highly expressed. Reaction efficiencies were used for relative quantification using the method as described by Pfaffl [30]. *Gapdh* was used as an internal reference gene. Primers used for qRT-PCR are listed in Additional file 2: Table S1.

#### Statistics

To compare gene expression data obtained with qRT-PCR, statistical analyses were performed using GraphPad Prism 5.0. Statistical significance was determined using a two-tailed Student’s *t*-test for comparison between pairs of means. *P* values <0.05 were considered to be statistically significant.

### Immunohistochemistry and morphometric analyses

At 24 h and 1 week after irradiation (0.0 or 1.0 Gy at E11), pregnant mice were sacrificed by cervical dislocation and embryos were dissected and processed for immunohistochemical analysis. Juvenile animals (4 weeks old, 0.0 or 1.0 Gy at E11) were processed similarly to study hippocampal neurogenesis. At least three animals were used in each test.

#### Tissue collection and staining

Embryos and juvenile brains were fixed overnight in 10% buffered formalin and dehydrated in a graded series of ethanol. Consequently, 4-μm-thick paraffin sections were cut. Before incubation in 3% H_2_O_2_ for 10 min, sections were dewaxed, rehydrated and heated in unmasking buffer. The primary antibodies used included the following: polyclonal rabbit anti-cleaved caspase-3 (1/200, Cell Signaling Technology, Danvers, MA, USA), polyclonal rabbit anti-GFAP (1/500, Dako, Carpinteria, CA, USA), polyclonal rabbit anti-Sox2 (1/500, Abcam, Cambridge, UK), polyclonal rabbit anti-DCX (1/500, Abcam, Cambridge, UK), polyclonal rabbit anti-Ki67 (1/600, Monosan, Uden, The Netherlands) and polyclonal rabbit anti-Iba1 (1/500, Wako Pure Chemical Industries, Osaka, Japan). Antibody-antigen complexes were visualised using a rabbit biotinylated secondary antibody. After incubation with avidin-biotin, the immunoperoxidase staining was visualised with the Vector NovaRED Substrate Kit (Vector Laboratories Inc., Burlingame, CA, USA). Immunohistochemical detection of the monoclonal mouse antibody against PCNA (1/80, Calbiochem, Germany) was performed using the HistoMouse-MAX kit (Invitrogen Corporation, Camarillo, CA, USA) according to the manufacturer’s instructions. Antibody-antigen complexes were visualised using a horseradish peroxidase secondary antibody (Dako North America Inc., Carpinteria, CA, USA) and the DAB chromogen system (Dako North America Inc., Carpinteria, CA, USA).

#### Visualisation and morphometric analyses

**Figure 2:**
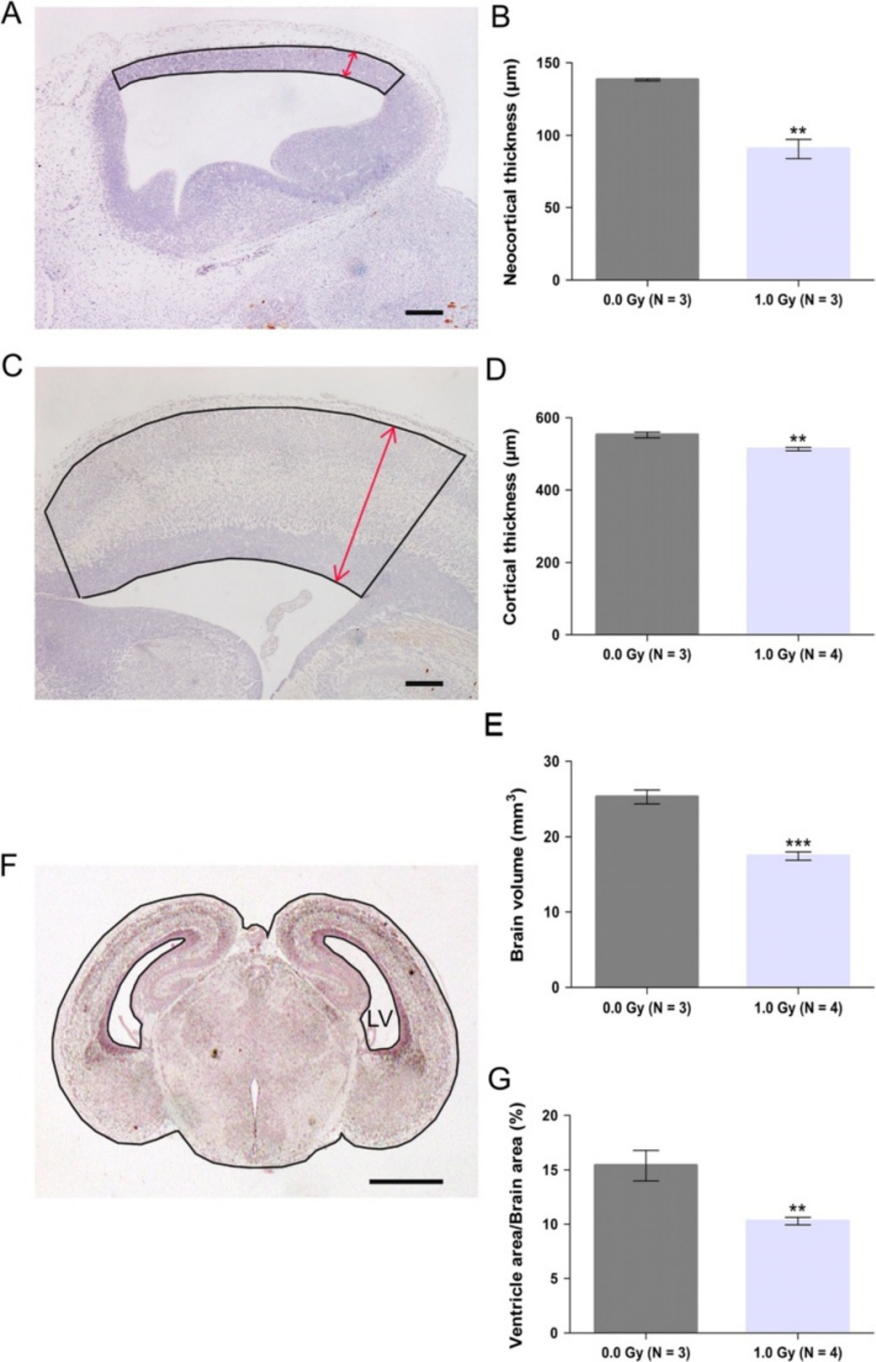
**Reduction in brain size, cortical thinning and smaller ventricles in the irradiated embryonic brain. (A, C, F)** Representative images are shown for the neocortex at 24 h after irradiation **(A)** and for the cortex **(C)** and the whole brain **(F)** at 1 week post irradiation with 1.0 Gy, with the indication of the lateral ventricle (LV) and the total brain area (outlined in **(F)**). **(B, D, E, G)** Morphometric measurements showed a reduction in neocortical thickness at 24 h **(B)** after radiation exposure. Furthermore, at 1 week post irradiation with 1.0 Gy of X-rays, the thickness of the cortex **(D)**, as well as the total brain volume **(E)** and the ventricle area **(G)**, was significantly reduced in the irradiated embryos. Data are presented as mean ± SEM. **P < 0.01, ***P < 0.001 for comparison with controls. The number of animals used per test is indicated in the graphs (*N*). Scale bar: 100 μm in **(A)** and **(C)**, 1,000 μm in **(F)**.

Thickness of the (neo)cortex: To determine the thickness of the neocortex at 24 h after irradiation and of the cortex at 1 week after irradiation, the average of 10 random measurements within the region of interest (outlined in Figure 2A,C) was calculated for one section per embryo. The imaging software NIS-Elements BR 4.00.05 (Nikon Instruments Europe B.V., Italy) was used.

Volumetric analysis of the brain: Serial coronal sections were sampled every 100 μm through all cerebral hemispheres in embryos at 1 week post irradiation. The area (Figure 2F) was measured in each section, multiplied by 100 μm (i.e. the distance between two successive sections) and summed, assuming lack of significant changes between subsequent sections.

Lateral ventricle area analysis: To standardise the sampling, the lateral ventricle area (Figure 2F) was measured in the section with the largest brain area and expressed as the percentage of the whole brain area.

**Figure 3:**
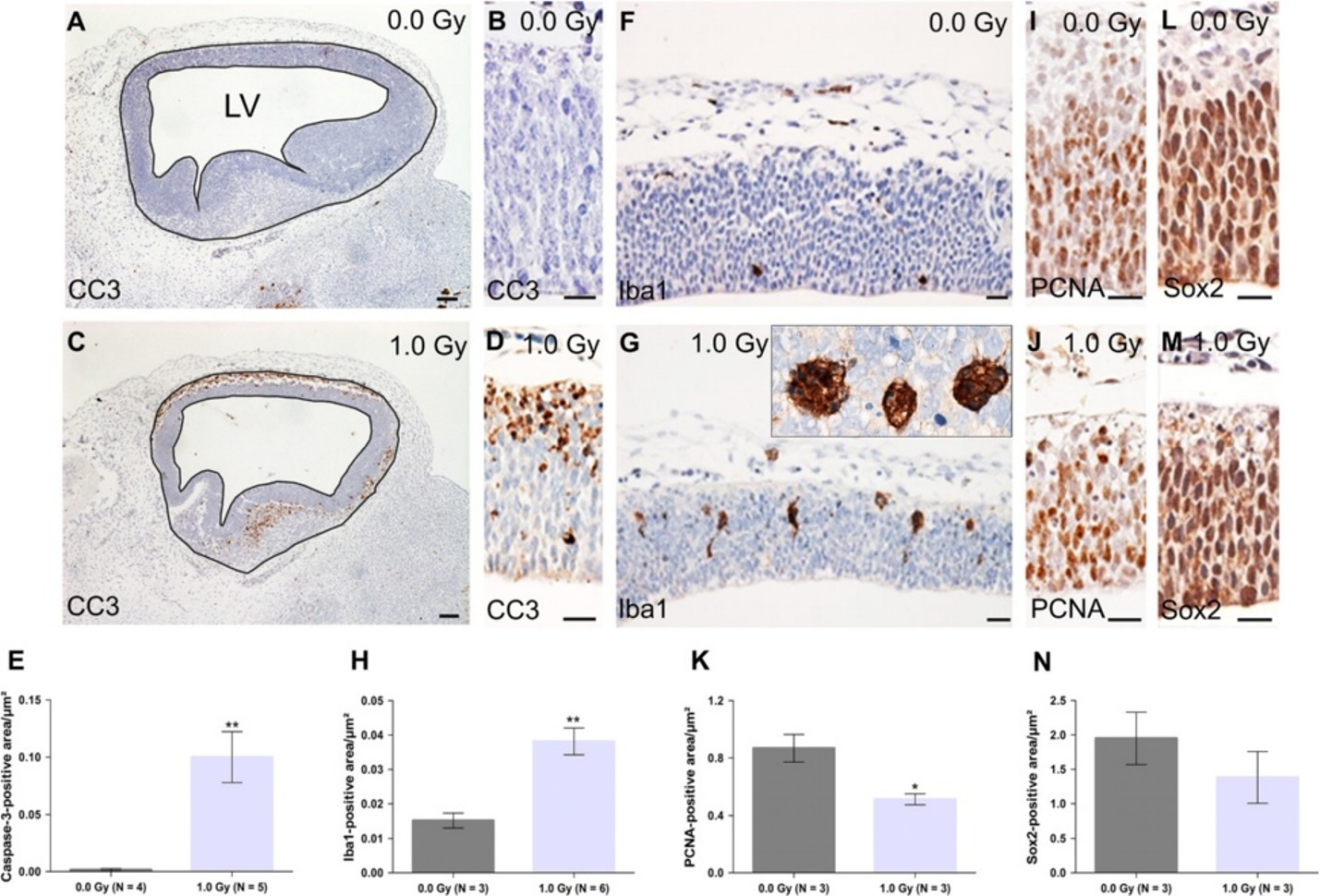
**Apoptosis, microglial activation and a decrease in PCNA-positive cells at 24 h after irradiation.** We quantified cleaved caspase-3 (CC3), Iba1, PCNA and Sox2 immunostainings in the embryonic brain of controls and 1.0-Gy-exposed mice. More specifically, we analysed changes in the region surrounding the lateral ventricle (LV) (as delineated in **(A)** and **(C)**) at 24 h after radiation exposure. **(A-E)** CC3 immunoreactivity showed a marked increase in apoptosis in the upper cortical layer in the 1.0-Gy-irradiated brains **(C, D, E)**, as compared to the control condition **(A, B, E)**. **(B)** and **(D)** are magnifications of the CC3 staining shown in **(A)** and **(C)**. **(F-H)** Iba1 staining revealed an increase in the Iba1-positive area following irradiation **(G, H)**, when compared to non-irradiated embryonic brains **(F, H)**. An enlargement of Iba1-positive microglial cells which engulf apoptotic nuclei is provided in the upper right corner in **(G)**. **(I-K)** A decreased PCNA-positive area was observed after 1.0-Gy radiation **(J, K)**, compared to non-irradiated controls **(I, K)**, while **(L-N)** no significant changes in Sox2-positive progenitor cells were observed in the irradiated brains **(M, N)**, as compared to control embryos **(L, N)**. Data are presented as mean ± SEM. *P < 0.05, **P < 0.01 for comparison with controls. The number of animals used per test is indicated in the graphs (*N*). Scale bar: 20 μm (100 μm in **(A)** and **(C)**).

Expression analysis in the embryo: For cleaved caspase-3 (CC3), Iba1, PCNA and Sox2 analyses, one section of the embryonic brain (at 24 h or 1 week after irradiation) was immunostained and imaged with HistoFAXS software (TissueGnostics, Austria) at a 10× magnification. Specific regions of interest (delineated in Figure 3A,C) were analysed with HistoQuest software (TissueGnostics, Austria) for automatic colour separation and quantification and staining was expressed as stained positive area per μm^2^.

**Figure 4:**
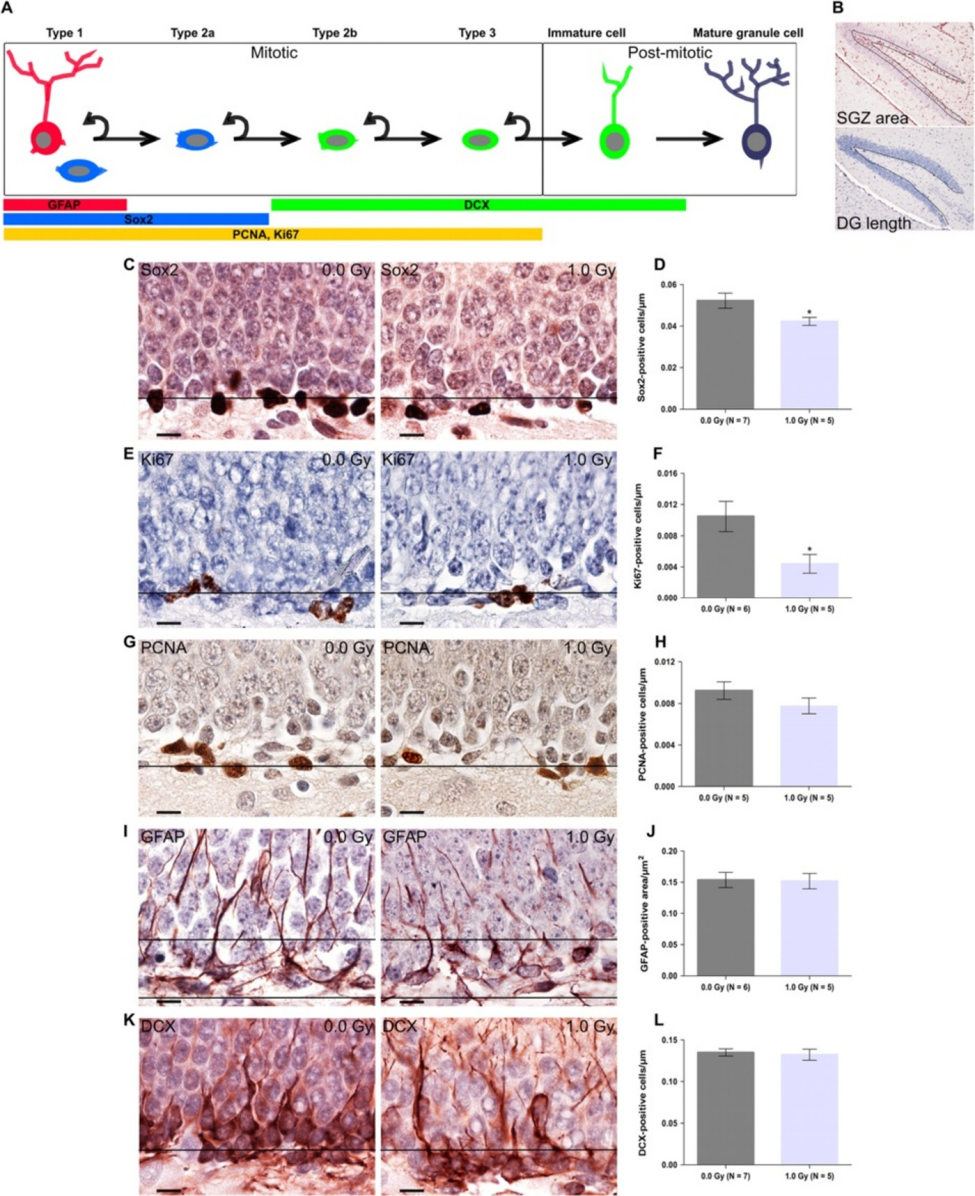
**Prenatal radiation exposure of 1.0 Gy at E11 impairs juvenile hippocampal neurogenesis. (A)** An overview of hippocampal neurogenic stages is shown, with the indication of immunohistochemical markers used in our study: Sox2, Ki67, PCNA, GFAP and DCX. **(B)** Quantification of these endogenous markers was performed using either the subgranular zone (SGZ) area (for GFAP) or the length of the dentate gyrus (DG) (for the other markers). **(C-L)** We showed a decrease in Sox2-positive **(C, D)** and in Ki67-positive **(E, F)** cells, but no change in PCNA **(G, H)**, GFAP **(I, J)** and DCX **(K, L)** expression when compared to controls. Data are presented as mean ± SEM. *P < 0.05 for comparison with controls. The number of animals used per test is indicated in the graphs (*N*). Scale bar: 10 μm.

Analysis of hippocampal neurogenesis: Cell quantification was performed on sagittal sections. The sections were collected starting from 500 μm to the midline and two or four non-overlapping sections from each hemisphere, representing the rostral/mid-hippocampus, were taken. For quantification of radial glial cells, GFAP-stained sections were imaged with HistoFAXS at a 10× magnification. The subgranular zone (SGZ) area of the dentate gyrus (DG; dashed line in Figure 4B, upper panel) was analysed with HistoQuest software for automatic colour separation and quantification. All images were analysed using identical software settings. The number of GFAP-positive cells was expressed as the fraction of the labelled area out of the total SGZ area. For Ki67, Sox2 and DCX quantification, the number of positive cells in the SGZ was expressed per DG length (μm) (dashed line in Figure 4B, lower panel), measured by the imaging software NIS-Elements BR 4.00.05.

#### Statistics

Statistical analyses were performed using GraphPad Prism 5.0. Statistical significance was determined using a two-tailed Student’s *t*-test for comparison between pairs of means. *P* values <0.05 were considered to be statistically significant.

## Results

### Exposure to X-rays at the onset of neurogenesis elicits a behavioural response in young adult mice dependent on the timing of irradiation

Male animals irradiated with different doses of X-rays (0.0, 0.2, 0.5 or 1.0 Gy) at selected time points of embryonic development (E10, E11 or E12) were subjected to a series of behavioural tests at the young adult age of 12 weeks. For all experimental groups, we analysed hippocampal-dependent spatial learning and memory in the MWM. Both the acquisition phase, composed of eight daily trial blocks, as well as a probe trial, performed at 4 days after the final acquisition trial block, were analysed. Furthermore, we measured circadian cage activity and examined motor ability using the accelerating rotarod test.

**Figure 5:**
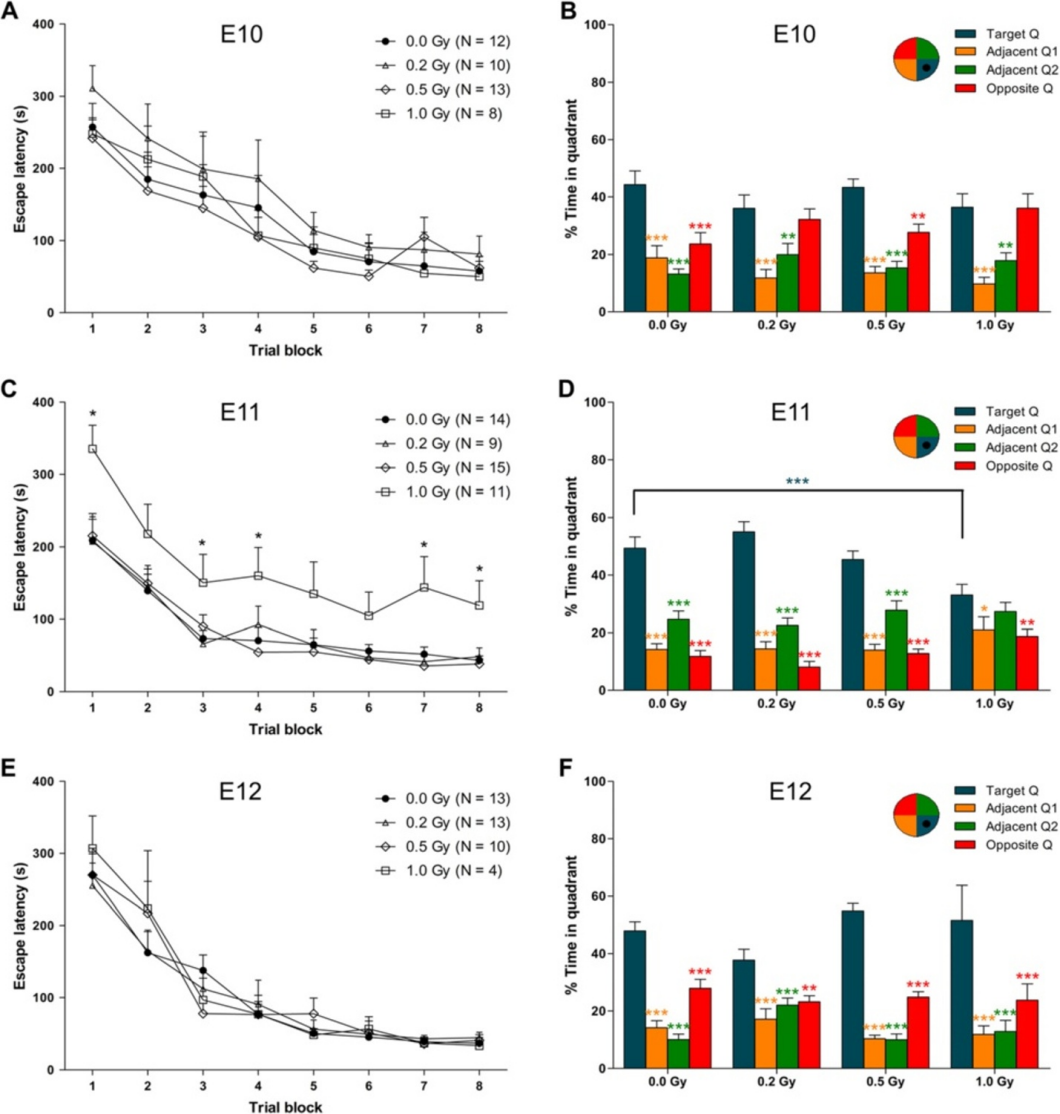
**Exposure to X-rays at E10, E11 or E12 compromises MWM learning in a timing-specific manner.** Results of the Morris water maze (MWM) acquisition trials and the probe trial, performed at 4 days after the final acquisition trial block, are shown for 12-week-old male mice, irradiated at E10 **(A, B)**, E11 **(C, D)** or E12 **(E, F)** with different doses of X-rays (0.0, 0.2, 0.5 or 1.0 Gy). **(A, C, E)** Escape latency during the MWM indicated that all mice improved their performance during the acquisition trial blocks, with no significant changes between sham-exposed animals and animals irradiated at E10 **(A)** or E12 **(E)**. Contrarily, mice exposed to 1.0 Gy at E11 **(C)** showed a consistently higher escape latency when compared to controls or animals irradiated with lower doses at the same age. **(B, D, F)** Probe trial results further revealed an inferior memory ability in the animals irradiated at E10 and E11, shown by a similar amount of time spent in the opposite quadrant as in the target quadrant at 0.2 and 1.0 Gy **(B)** or a significant difference in time spent in the target quadrant at 1.0 Gy when compared to controls (blue asterisks in **(D)**). Animals exposed to radiation at E12 **(F)** did not differ in target preference. Orange asterisks in **(B, D, F)** indicate significant differences in time spent in the adjacent Q1 quadrant as opposed to the target quadrant for each experimental group. Similarly, green and red asterisks point out significant differences in time spent in the adjacent Q2 and opposite quadrant, respectively. Data are presented as mean + SEM. *P < 0.05, **P < 0.01, ***P < 0.001. The number of animals used is indicated in the acquisition graphs (*N*).

The performance of all experimental groups during the MWM acquisition trials was improved (effect of trial block on escape latency: *P* < 0.001). Irradiation of pregnant mice at E10 did not cause deviations in MWM acquisition when compared to sham-irradiated controls (effect of irradiation dose on escape latency: *P* = 0.308, Figure 5A). However, animals irradiated with 0.2 or 1.0 Gy of X-rays did show a lack of preference for the target quadrant during the probe trial, as shown by a similar amount of time spent in the opposite quadrant as in the target quadrant (Figure 5B). This might be suggestive of an impaired consolidation of reference memory without spatial learning defects [31]. Interestingly, spatial learning was affected in animals exposed to radiation at E11. Here, irradiation with 1.0 Gy of X-rays resulted in deviating learning curves during the MWM acquisition as compared to mice exposed to lower doses and controls (effect of irradiation dose on escape latency: *P* < 0.001, Figure 5C). Although these mice spent more time in the target quadrant than in the opposite quadrant during the probe trial, the overall swim pattern was very different from that in control animals (Figure 5D). Indeed, animals irradiated with 1.0 Gy at E11 did not spend significantly more time in the target quadrant versus the adjacent Q2 quadrant while the amount of time spent in the target quadrant was significantly reduced compared to controls. Thus, both spatial learning and memory were affected in these mice. Finally, in mice irradiated at E12, we did not observe differences in MWM acquisition (effect of trial block on escape latency: *P* = 0.981, Figure 5E) nor in target quadrant preference (Figure 5F). Finally, control mice of the E11 irradiation group differed mildly in adjacent Q2 and opposite quadrant preference when compared to controls from E10 and E12, possibly attributed to inter-group variability. Yet, no difference in target quadrant preference could be observed, indicating that all mice learned the Morris water maze paradigm and could be used as appropriate controls. In all, spatial learning and long-term memory seem most affected by an irradiation dose of 1.0 Gy at E11.

**Figure 6:**
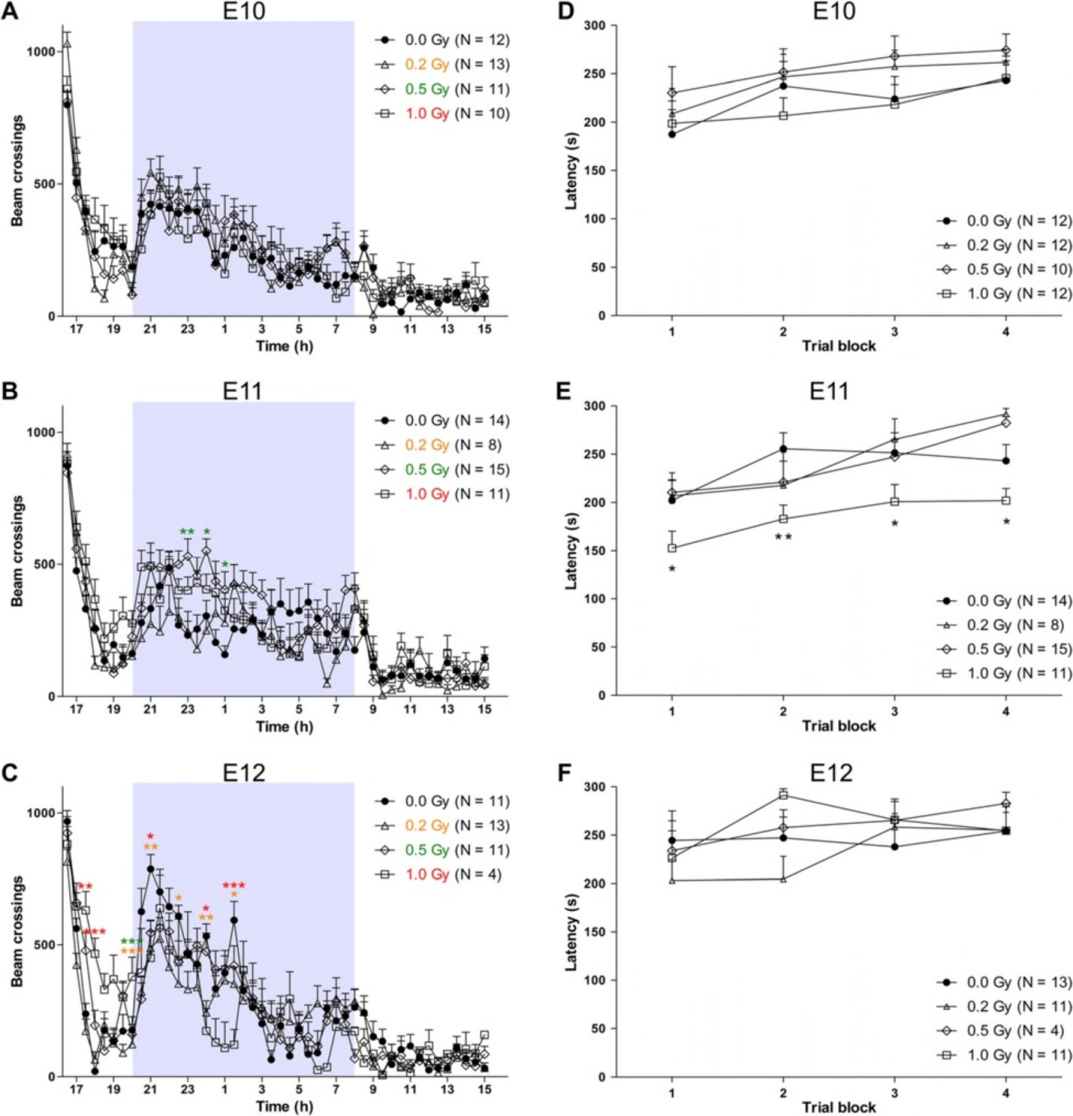
**Cage activity and motor behaviour are affected time-dependently by irradiation at the onset of neurogenesis.** Results of the 23-h horizontal cage activity task **(A, B, C)** and of the accelerating rotarod test **(D, E, F)**, both performed in 12-week-old male mice, irradiated at E10 **(A, D)**, E11 **(B, E)** or E12 **(C, F)** with different doses of X-rays (0.0, 0.2, 0.5 or 1.0 Gy). **(A, B, C)** Recordings for each 30-min time interval revealed an unchanged circadian cage activity profile in animals irradiated at E10 **(A)**. Mice exposed to 0.5 Gy at E11 **(B)** showed a mild hyperactivity during the dark phase of the circadian cycle. In animals exposed to radiation at E12 **(C)**, a nocturnal hyperactivity was also observed, already from a dose of 0.2 Gy onwards, and was accompanied by a decreased horizontal activity during the light phase, i.e. between 16 and 20 h. Orange asterisks represent significant differences in mice irradiated with 0.2 Gy, green asterisks in mice irradiated with 0.5 Gy and red asterisks in mice irradiated with 1.0 Gy, all compared to non-irradiated controls. **(D, E, F)** Performance on the accelerating rotarod apparatus was strongly affected in the group of 1.0-Gy-irradiated mice at E11 **(E)** during each of the four trials as compared to all other conditions (indicated as reduced latency values). Animals exposed to radiation at E10 **(D)** or E12 **(F)** did not display a changed latency in the accelerating rotarod. Data are presented as mean + SEM. *P < 0.05, **P < 0.01, ***P < 0.001 for comparison with controls. The number of animals used per test is indicated in the graphs (*N*).

In a second test, we analysed 23-h ambulatory horizontal cage activity. When irradiated at E10, no alterations in circadian rhythm were detected between control and irradiated mice (effect of irradiation dose on beam crossings: *P* = 0.975, Figure 6A), whereas animals exposed to 0.5 Gy of X-rays at E11 did show mild hyperactivity, mainly observed during the dark phase, in comparison to control animals (effect of irradiation dose on beam crossings: *P* = 0.013, Figure 6B). Yet, this hyperactivity was not observed in animals exposed to a higher dose of 1.0 Gy or a lower dose of 0.2 Gy at E11. Finally, there was no significant difference in overall 23-h spontaneous cage activity between mice that had been sham-irradiated or X-irradiated at E12 (effect of irradiation dose on beam crossings: *P* = 0.232). However, at this embryonic age, *post hoc* testing revealed hyperactivity of all the irradiated groups during the light phase while showing hypoactivity during the dark phase (Figure 6C). Strikingly, when looking into more detail at activity patterns during the dark phase, we could show alterations in nocturnal activity at all three irradiation stages (two-way RM ANOVA, effect of irradiation dose on beam crossings during the night: *P* = 0.014, *P* < 0.001 and *P* < 0.001 for E10, E11 and E12, respectively). Altogether, we found an effect of prenatal irradiation on cage activity patterns, with the most pronounced changes occurring after X-ray exposure at E12.

Lastly, in the rotarod test, we did not observe significant differences in the latency to fall of the rod in mice irradiated at E10 (effect of irradiation dose on latency: *P* = 0.233, Figure 6D) or E12 (effect of irradiation dose on latency: *P* = 0.112, Figure 6F). On the other hand, mice exposed to 1.0 Gy of radiation at E11 did show a significant motor defect (effect of irradiation dose on latency: *P* = 0.006, Figure 6E). Hence, motor coordination and equilibrium was only affected by an irradiation dose of 1.0 Gy at E11, the developmental time point and dose at which irradiation also induced the most deviant spatial learning behaviour.

In conclusion, radiation exposure at the onset of neurogenesis clearly affected long-term neurobehavioural characteristics, which depend on the day of exposure. For further experiments, we aimed at investigating the effects of prenatal irradiation on the brain using a multidisciplinary approach, including MRI and MRS, DTI, gene expression profiling, histology and immunohistochemistry. Because of obvious constraints for such a thorough analysis, we focused only on mice irradiated with 1.0 Gy at E11 for the remainder of the study because these mice showed the clearest defects in behaviour. Moreover, it has previously been shown that the neuronal precursor pool at E11 is critical for later brain size [32], a feature which is often affected by prenatal radiation exposure [33, 34].

### Microstructural alterations in multiple brain regions of animals irradiated at E11

**Figure 7:**
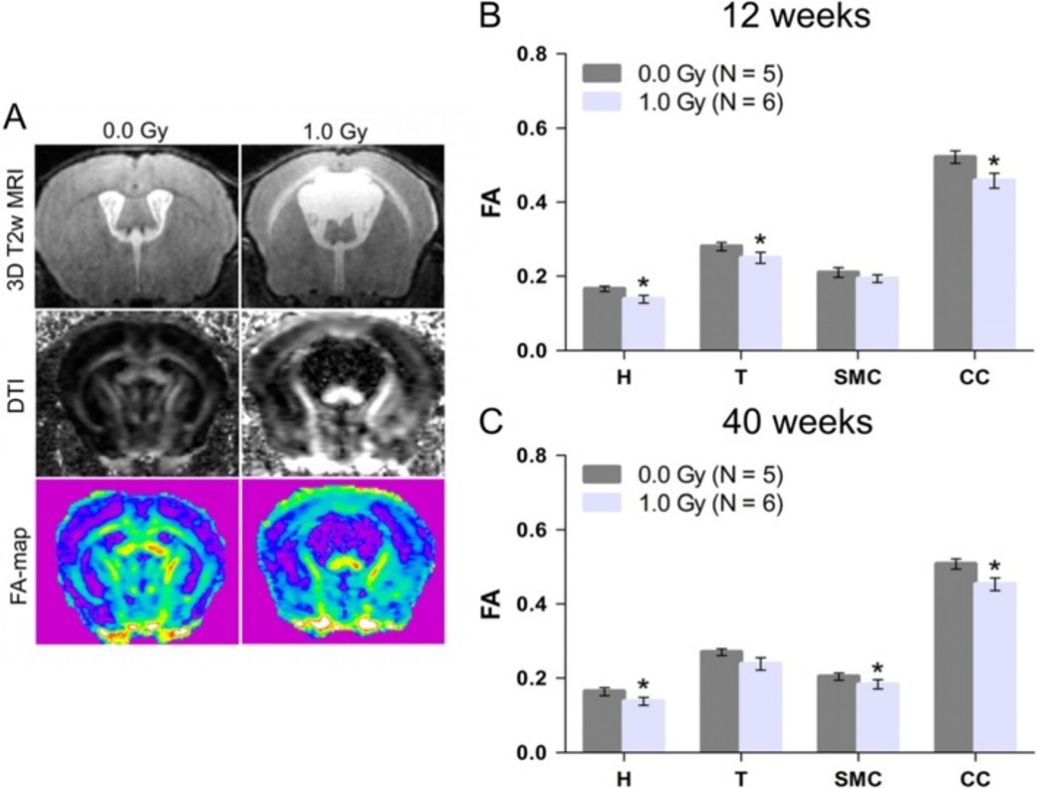
**Microstructural changes in multiple brain regions of mice exposed to 1.0 Gy at E11. (A)** Representative anatomical 3D T2-weighted MR images are shown, in combination with the associated diffusion tensor imaging (DTI) scans and corresponding fractional anisotropy (FA) maps. FA values were analysed for both animal groups in the hippocampus (H), thalamus (T), sensory-motor cortex (SMC) and corpus callosum (CC). **(B)** At 12 weeks of age, DTI scans revealed reduced FA in all regions, except for the SMC. **(C)** A similar trend was noted at 40 weeks after birth, with significantly decreased FA in the H, SMC and CC. Data are presented as mean ± SEM. *P < 0.05 for comparison with controls. The number of animals used per test is indicated in the graphs (*N*).

To investigate possible causes for the observed long-term behavioural effects after prenatal irradiation at E11, we performed *in vivo* DTI in animals at a young adult (12 weeks old) and adult (40 weeks old) age. The FA of the average water diffusion in DTI represents anisotropy within axons, providing a useful tool to study central nervous system connectivity. In addition, it is highly sensitive to both white and grey matter microstructural changes, stressing its suitability to detect neuropathologies and variations in microstructural composition or tissue architecture [35]. We measured FA (Figure 7A) in defined brain regions, such as the hippocampus, thalamus, sensory-motor cortex and corpus callosum (Additional file 1: Figure S1C). When compared to controls, lower FA values were observed in the hippocampus, thalamus and corpus callosum, but not in the sensory-motor cortex of the irradiated animals at the age of 12 weeks (Figure 7B). In adult mice, reduced FA values were still detected in the hippocampus and corpus callosum, but no longer in the thalamus. Interestingly, at this later stage, FA values in the sensory-motor cortex were now also found to be decreased (Figure 7C).

Altogether, we observed reduced FA in various brain regions of both young adult and adult mice irradiated at E11 with 1.0 Gy, indicative of microstructural changes. A significantly persistent decrease in FA was noted in the hippocampus and corpus callosum. However, since FA is very sensitive, but fairly non-specific [35], other measures are necessary to provide a conclusive cause for the alterations observed.

### Irradiation-induced cortical depletion of the neuronal marker NAA

In the 40-week-old animals, we also calculated metabolite concentrations from localised MR spectra of a cortical and subcortical (striatal) region (Figure 1A,B). This revealed significantly lower concentrations of the nervous system-specific metabolite NAA in the cortex of the irradiated animals (Figure 1C), indicative of a pathological loss of neurons [36, 37]. None of the other metabolite concentrations examined differed between the two conditions (Additional file 3: Table S2).

In conjunction with the DTI results, we thus observed prenatal radiation-induced defects in both the cerebral cortex and the hippocampus. For this reason, we focused on those two brain regions for further analyses.

### Exposure to radiation at E11 induces a rapid but transient p53-mediated transcriptional response

The long-term effects of *in utero* exposure to ionising radiation on the function and structure of the brain are complex and may depend, at least partially, on the early molecular response to stress signals (reactive oxygen species, DNA damage) elicited by irradiation. In order to better understand these early effects, we analysed genome-wide changes in gene expression of the whole brain both at 2 and 24 h after exposure of E11 mouse embryos to different doses of X-rays (0.0, 0.1, 0.2, 0.5 or 1.0 Gy).

**Figure 8:**
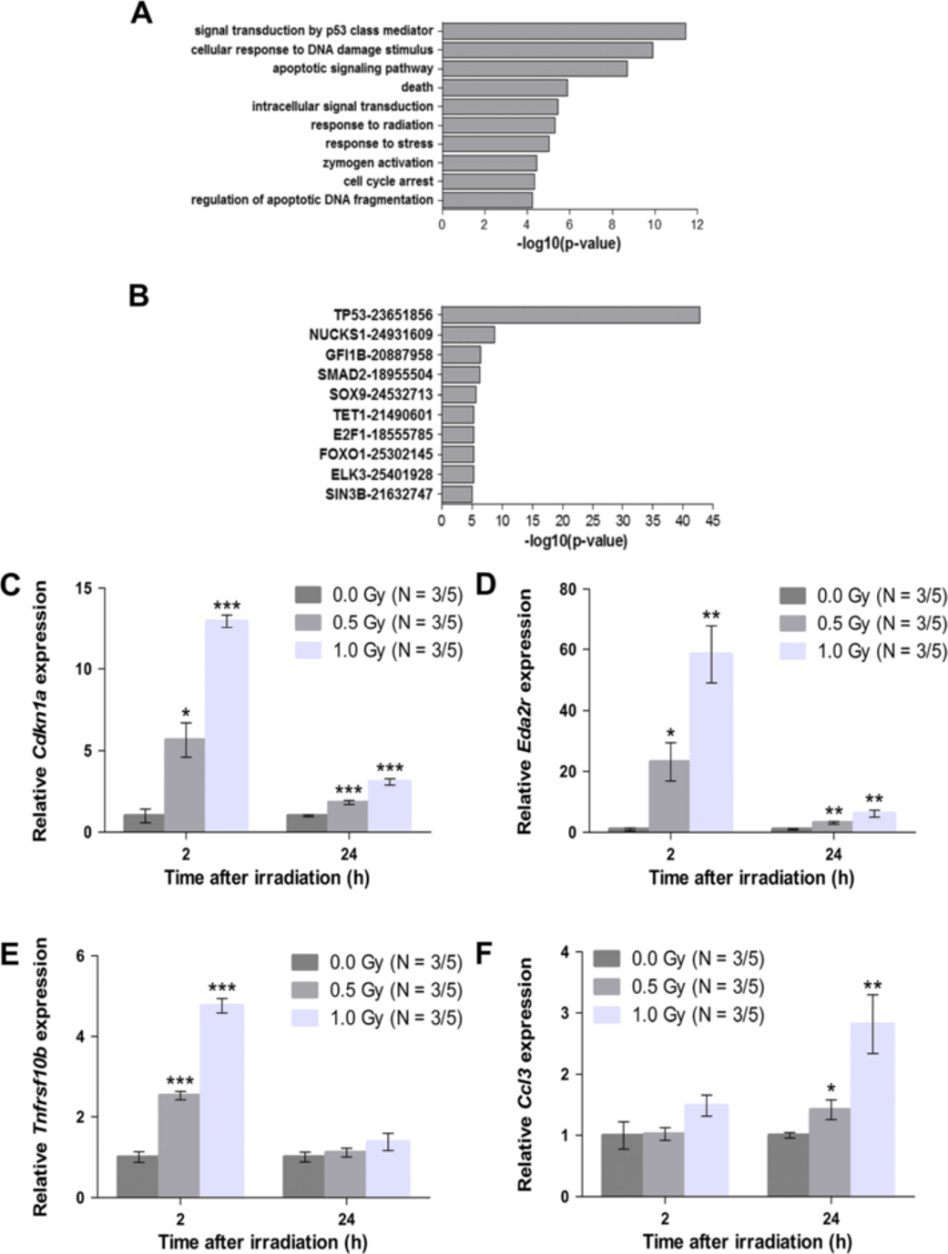
**An irradiation-induced early p53-mediated response is associated with the activation of the pro-inflammatory gene** ***Ccl3*** **. (A, B)** Gene Ontology (GO) and transcription factor binding site enrichment among differentially expressed genes in the mouse embryonic brain 2 h after exposure of the E11 pregnant mother to 1.0 Gy of X-rays. **(A)** Top 10 GO enrichment terms among differentially expressed genes at 2 h after irradiation showed a main involvement of the classical p53-regulated DNA damage response early after irradiation. **(B)** A chromatin immunoprecipitation (ChIP) enrichment analysis was employed to predict transcriptional regulators of differentially expressed genes 2 h after irradiation. **(C-F)** Quantitative reverse transcriptase PCR experiments were applied on a set of genes to confirm microarray findings: p53 targets (*Cdkn1a* **(C)**, *Eda2r* **(D)** and *Tnfrsf10b* **(E)**) and *Ccl3* **(F)**. Data are presented as mean ± SEM. *P < 0.05, **P < 0.01, ***P < 0.001 for comparison with controls. The number of animals used per test is indicated in the graphs (*N*/*N* for 2 h/24 h after irradiation).

For the earliest response, we found 41 genes that were differentially expressed after irradiation, with most genes showing a dose-dependent increase in expression. Functional enrichment analysis showed that these genes are mainly involved in the classical p53-regulated DNA damage response, i.e. genes involved in cell cycle arrest (e.g. *Ccng1*, *Cdkn1a*), DNA repair (e.g. *Polk*, *Ercc5*) and apoptosis (e.g. *Bax*, *Bbc3*, *Tnfrsf10b*) (Figure 8A). Further evidence for the importance of p53 in mediating these early gene expression changes came from a search in the ChIP enrichment analysis database [29]. Indeed, we found that p53 was by far the most significantly enriched transcription factor predicted to regulate these genes (Figure 8B). Other examples of predicted regulators of the gene expression changes we observed after 2 h were E2f1, which is known to initiate pro-apoptotic responses to DNA damage [38], and Smad2, which was recently shown to be localised to radiation-induced double-strand breaks and was proposed to cooperate with p53 to regulate the DNA damage response [39].

At 24 h after radiation exposure, only three genes (*Eda2r*, *Ccl3* and *Nhlrc1*) were identified as being differentially expressed in the irradiated embryos, of which the pro-apoptotic p53 target gene *Eda2r* was also differentially expressed after 2 h. This indicates that the early p53-mediated transcriptional response was only transiently activated after radiation exposure. Interestingly, for *Ccl3*, known to be involved in inflammation [40, 41], we could observe a dose-dependent induction of gene expression, which might be suggestive of a radiation-induced inflammatory response.

To validate some of these results, we performed qRT-PCR on a selection of genes. p53 targets (*Cdkn1a*, *Eda2r* and *Tnfrsf10b*) showed a strong and dose-dependent increase after 2 h, which was either attenuated (*Cdkn1a*, *Eda2r*) or completely abrogated (*Tnfrsf10b*) at 24 h (Figure 8C–E). In contrast, the pro-inflammatory gene *Ccl3* showed a dose-dependent increase only after 24 h (Figure 8F). Together, our gene expression data thus suggest an early radiation-induced p53-dependent response that might be accompanied or followed by an inflammatory response at 24 h after irradiation.

### Radiation-induced early- and long-term effects: an eye on the cortex

As previously mentioned, we detected both radiation-dependent reduced FA and decreased NAA concentrations in cerebral cortical regions of the adult brain. In order to identify possible causes for these observations, we next focused on the cortical region for more in-depth follow-up experiments. To this end, we used immunohistochemistry and morphometric analyses to investigate brain morphology and protein expression at 24 h and 1 week after irradiation at E11 with 1.0 Gy. Additionally, postnatal brain and cortical morphology were assessed using MRI.

First, to further validate the gene expression results, we analysed apoptosis and microglial inflammation in the embryonic neuroepithelium surrounding the lateral ventricle, encompassing the neocortex (outlined in Figure 3A), using CC3 and Iba1 immunohistochemistry, respectively. At 24 h after irradiation, we detected a widespread 64-fold increase in apoptosis in mice irradiated with 1.0 Gy (Figure 3A–E). Remarkably, we observed a strong expression of CC3 in the outer layers of the neocortex, whereas almost no staining could be observed in the inner ventricular zone. This indicates that apoptosis primarily affected differentiating neurons, rather than progenitor cells. Moreover, the activation of neuroinflammation in the irradiated embryonic brain was confirmed by a clear increase in total Iba1-positive area 24 h after irradiation (Figure 3F–H). Visual inspection of the immunostaining showed that there was an increase both in the absolute number and in the size of the Iba1-positive cells in the irradiated embryonic brains compared to controls. Importantly, similar to the localisation of CC3-positive cells and in concordance with the well-recognised phagocytic function of microglia in the brain [42, 43], the Iba1 staining was mainly found in the outer layers of the neocortex, where Iba1-positive microglial cells were shown to engulf whole cells (magnification in Figure 3G). Furthermore, progenitor cell proliferation was significantly decreased in the irradiated embryos, as shown by a reduced expression of the cell cycle marker PCNA (Figure 3I–K). In contrast, the amount of Sox2-positive progenitor cells was not affected (Figure 3L–N). Finally, and presumably related to the increased apoptosis and decreased proliferation, the thickness of the neocortex was significantly reduced at 24 h after radiation exposure in comparison to unexposed controls (Figure 2A,B).

At 1 week post irradiation, apoptosis and proliferation were restored to control levels in the cerebral cortex (Additional file 4: Figure S2), while the difference in cortical thickness was still apparent (Figure 2C,D). Furthermore, the overall brain volume of these irradiated animals was decreased (Figure 2E). Notably, the area of the lateral ventricles, normalised for brain area, was also significantly reduced in the irradiated mice as compared to non-irradiated animals (Figure 2F,G).

**Figure 9:**
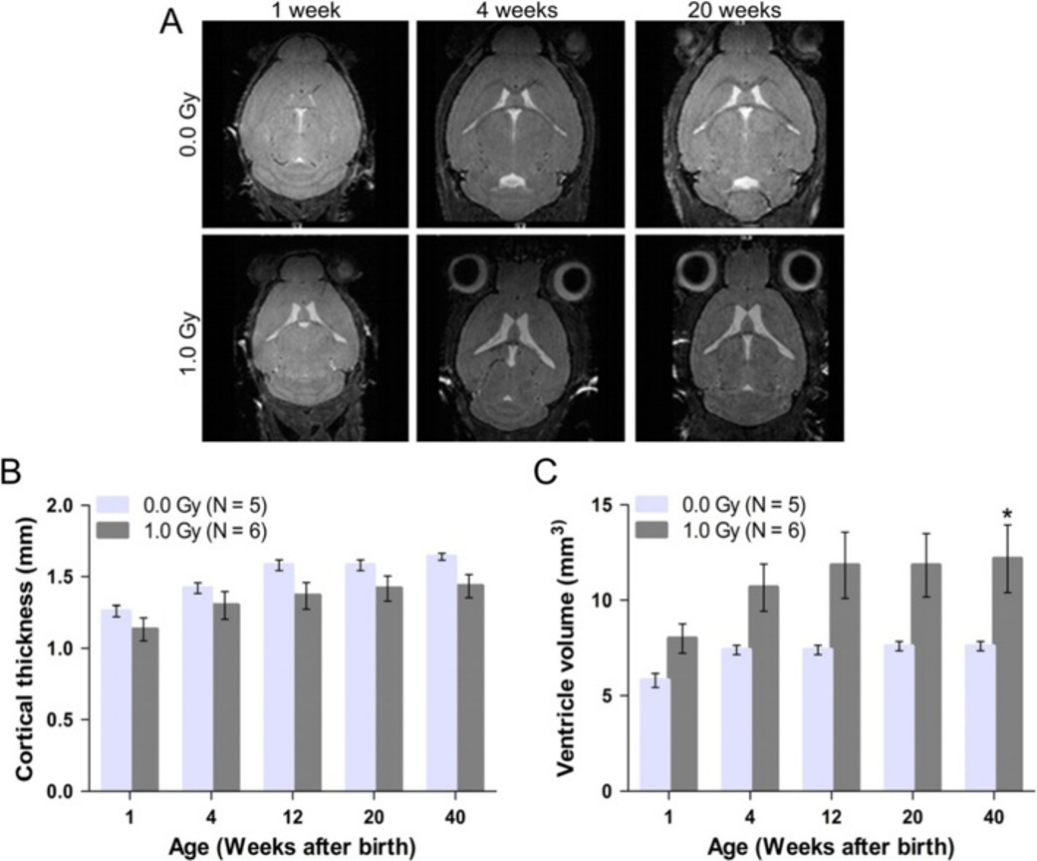
**Animals exposed to 1.0 Gy at E11 display persistent changes in brain anatomy. (A)** Representative anatomical 3D T2-weighted MR images of control animals and mice irradiated with 1.0 Gy at E11 at 1, 4 and 20 weeks after birth. **(B)** Quantitative analysis of these MR images demonstrated that the cortical thickness of the irradiated mice was mildly decreased, although not statistically significant. **(C)** At similar time points, these animals showed dilated ventricles, which was significant in 40-week-old animals. Data are presented as mean ± SEM. *P < 0.05 for comparison with controls. The number of animals used per test is indicated in the graphs (*N*).

To test whether these radiation-induced morphological differences were still evident postnatally, we subjected animals to 3D T2-weighted MRI (Figure 9A). For this purpose, the non-invasive MRI procedure allowed us to image mice at different time points during their lifetime, ranging from postnatal/juvenile (1 and 4 weeks after birth) until young adult (12 weeks after birth) and adult (20 and 40 weeks after birth) stages. These sequential analyses demonstrated mild differences in brain anatomy between control and irradiated mice. More specifically, while the irradiated animals did not show a reduction in cortical thickness (Figure 9B), a significant enlargement of the ventricles was noted at 40 weeks of age (Figure 9C). Hence, in the cerebral cortex, early radiation-induced defects may eventually lead to late-occurring morphological alterations.

### Radiation-induced early- and long-term effects: an eye on the hippocampus

As mentioned before, prenatal irradiation at E11 resulted in defects in spatial learning and memory in the MWM test, suggesting impaired hippocampal function. Since early postnatal radiation exposure has repeatedly been shown to negatively affect hippocampal neurogenesis [44–46], we decided to investigate neurogenesis in the developing and juvenile hippocampus.

At 1 week post irradiation, no difference was found in CC3, PCNA or Sox2 immunoreactivity (Additional file 5: Figure S3). Next, we investigated radiation-induced changes in the cellular composition of the juvenile hippocampal DG. In mice, development of the DG is completed by the fourth postnatal week, the time at which precursors settle in the SGZ and start to generate neurons throughout life, a process referred to as hippocampal neurogenesis [47]. Postnatal hippocampal neurogenesis is a multistep process that recapitulates all stages of neuronal development, however in a mature central nervous system. Type 1 radial stem cells, exhibiting astrocytic properties, express GFAP, whereas Sox2 immunoreactivity can be found, besides in GFAP-positive radial stem cells, in horizontal type 1 stem cells. These stem cells give rise to transient amplifying precursors, of which the type 2a cells still express Sox2 [48]. Upon differentiation and migration to the granule cell layer, immature neurons express DCX [49]. Post-mitotic mature granule neurons further integrate functionally in the DG. We analysed neurogenesis in the DG in 4-week-old mice, corresponding to a juvenile stage characterised by a high rate of neurogenesis [50, 51]. Hereto, we employed immunohistochemistry for Sox2, Ki67, PCNA, GFAP and DCX (Figure 4A). Immunoreactivity was assessed by measuring the positively stained area, normalised with respect to the SGZ area for GFAP (upper panel in Figure 4B) or by counting the amount of positive cells, normalised with respect to the length of the DG for all other markers (lower panel in Figure 4B). A significant reduction in Sox2-positive cells (Figure 4C,D) and in Ki67 immunoreactivity (Figure 4E,F) was found in the irradiated versus control animals, while the number of PCNA-positive cells (Figure 4G,H), GFAP-positive stem cells (Figure 4I,J) and immature DCX-positive neurons (Figure 4K,L) remained unchanged.

Thus, we can conclude that radiation exposure of the E11 embryo impairs hippocampal neurogenesis through a specific depletion of proliferative and Sox2-positive cells at a juvenile stage. Given that Sox2 predominantly marks type 1 radial and horizontal stem cells and type 2a intermediate precursors and the fact that no changes were observed in GFAP immunoreactivity, which specifically stains radial stem cells [48, 52], we suggest that the horizontal stem cells and/or fast proliferating intermediate cells are suppressed as a result of E11 irradiation.

## Discussion

Several epidemiological studies have provided evidence for neurological aberrations in children prenatally exposed to ionising radiation [4]. For example, *in utero* radiation exposure after the bombings of Hiroshima and Nagasaki resulted in a higher incidence of small head size, mental retardation, lower IQ values and seizures. Notably, this was only apparent when they had been exposed between gestational weeks 8 and 15 and to a lesser extent between weeks 16 and 25 [6], indicating that the timing of irradiation is very crucial. Nowadays, pregnant women can still be exposed to sources of radiation during medical diagnostic procedures and/or treatment. Radiotherapy, for instance, is sometimes essential to treat cancer patients during pregnancy, but little information exists on the long-term outcome of the exposed embryos or fetuses [9]. Importantly, the time points used in our study are characterised by strongly polarised symmetrical cell divisions in the ventricular zone in order to generate neurons and do not correspond to the radiosensitive gestational period of weeks 8 to 15 in humans [13, 53], in which neuronal migration and differentiation primarily occur [10]. This work thus proposes an expansion of the radiosensitivity period, starting even earlier during the course of central nervous system development and resulting in the induction of adult behavioural deficits. A previous study by Baskar and Devi [54] also assessed murine sensitivity to *in utero* radiation by using behavioural tests in the young adult offspring. Similarly, these authors found a higher susceptibility to ionising radiation during the earliest stages of brain development. However, in their study, the effects were not as distinct between the gestational ages, with only minor differences observed between both mice irradiated at E11.5, E12.5, E14.5 and E17.5. This discrepancy might be explained by the fact that their findings were based on a completely different set of behavioural tests, including the open field for locomotor assessment and the hole board and active avoidance to test learning and memory [54]. Furthermore, in their study, mice of both sexes were anaesthetised in order to irradiate with a higher dose rate than in our experiments. The highest dose used was 0.5 Gy and experiments were performed in Swiss albino mice, possibly all factors contributing to the non-consistent behavioural results [54]. Here, most obvious behavioural aberrations were noted after irradiation at E11 with a dose of 1.0 Gy, which is why we focused on this condition for further analyses. Intriguingly, the time dependency observed in our study may be due to the disruption of particular cell populations with distinct functions and positioning in the adult brain, which would then also affect different behavioural aspects [19].

*In utero* exposure to ionising radiation generally induces a classical p53-mediated response, resulting in extensive neuronal cell death, depending on the dose [55–58]. Indeed, we also observed this transient p53-dependent apoptotic response after irradiation with a moderate dose of X-rays. Of interest, the CC3 staining pattern at 24 h after exposure illustrates that most cell death occurs in the outer layers of the neocortex, constituted of differentiating neurons, which is contradictory to previous research in which neural stem or progenitor cells undergo apoptosis in response to irradiation [55]. This finding is also contradictory to one of the dogmas in radiation biology known as the law of Bergonie and Tribondeau, which states that the radiosensitivity of cells is related to their reproductive capacity [59]. Therefore, proliferating cells in the developing brain have always been considered more radiosensitive compared to post-mitotic cells [55, 60]. Moreover, it has been shown that the DNA damage-induced apoptotic response in differentiating migrating neurons is p21-dependent, whereas stem and progenitor cells repress p21 expression [61]. In accordance with these findings, we also observed a dose-dependent increase in the expression of p21 (*Cdkn1a*) at 2 and 24 h after irradiation, which may then trigger apoptosis in differentiating neurons.

Irrespective of which cells undergo cell death, the presence of apoptotic cells probably relates to an increase in Iba1-positive microglial cell size at 24 h after irradiation in the developing neocortex. This, in combination with the appearance of multinucleated microglial cells, indicates phagocytosis of multiple dead cells by activated microglia. Furthermore, we also observed an increase in Iba1 cell number at 24 h after exposure, indicating inflammatory processes. This was further substantiated by a dose-dependent induction of *Ccl3*, a pro-inflammatory cytokine that plays a critical role in radiation-induced inflammation [40, 41]. Altogether, these gene expression and immunohistochemical data suggest that an inflammatory response to prenatal radiation, involving the activation of microglia, is associated with radiation-induced neuronal cell death. Interestingly, in the prenatally irradiated adult animals, we also found evidence for persistent neuroinflammation and astrogliosis, based on DTI and MRS parameters. The combination of reduced FA and NAA, both apparent in the cortex at 40 weeks of age, is indeed indicative of inflammatory processes and astroglial activation [36, 62]. Yet, additional experiments are needed to assess whether this inflammation and/or astrogliosis may contribute to the observed abnormalities.

Further in-depth analysis revealed a reduction in PCNA staining in the area surrounding the lateral ventricle at 24 h after irradiation. PCNA is highly expressed in dividing cells and its immunoreactivity is detectable in late G1- and S-phase [63]. The reduction in PCNA staining might thus reflect an irradiation-induced block in the cell cycle since gene expression analysis also revealed an upregulation of genes involved in cell cycle arrest.

Radiation-induced apoptosis in the preplate, subsequent microglial cell activation and a decreased proliferation in the ventricular zone of the developing cortex logically result in cell loss, which is in line with the observation of a reduced cortical thickness at 24 h and 1 week after irradiation. However, as opposed to previous studies demonstrating postnatal thinning of the cortex in prenatally irradiated animals [34, 64], we could not demonstrate such a significant difference after birth. Nevertheless, we noted a decline in concentration of cortical NAA, a key MRS marker for vital neuroaxonal tissue, in 40-week-old animals, indicative of a pathological loss of neurons or atrophy [36, 37]. Finally, such a decrease in cellular density might also explain the dilation of the ventricles at 40 weeks after birth. In fact, prenatal apoptosis has already been linked to severe postnatal hydrocephalus [65], further substantiating the early- and late-term consequences shown in this study. Still, additional studies will be required to explain the reduction in ventricular size at 1 week post irradiation and a dilation of the ventricles after birth. Possibly, the transient reduction in lateral ventricle size may be a direct consequence of the reduced cell proliferation, considering that cell divisions are required for proper ventricular development [66] and that an interaction exists between the production of embryonic cerebrospinal fluid and proliferation, although mechanisms remain undiscovered [67].

At 4 weeks after birth, in the DG, the level of Sox2-positive cells, but not that of GFAP-positive radial stem cells, was significantly reduced in irradiated mice. Analysis of hippocampal proliferation at this juvenile age revealed no changes in PCNA-positive cells. Given that PCNA is only expressed in G1- and S-phase cycling cells [63], Ki67 immunostainings were performed to label dividing cells in all cell cycle phases [52]. This led to the observation of a decreased overall proliferation in the irradiated hippocampus compared to non-irradiated controls. Further investigation is desired to unravel whether the decline in proliferation and in Sox2-positive stem cells, by depleting the stem cell pool, has consequences for later maturation and synaptic functional integration of these neurons. For instance, immunohistochemistry for synaptic markers and electrophysiological recordings might be of great interest considering that an impairment of hippocampal neurogenesis is strongly linked to cognitive defects [68]. To our knowledge, only one study has previously analysed radiation-induced effects on hippocampal neurogenesis, by using BrdU labelling of S-phase cells in the hippocampus of 12-week-old animals. These authors did not find a difference in neurogenesis between non-irradiated and 1.0-Gy-irradiated E16 rats, although MWM screening showed a deficiency in short-term, but not long-term, spatial memory [69]. Notably, and opposed to the study of Tomasova et al*.*, we analysed neurogenesis in juvenile mice, of which the observed neurogenic decrease might have more serious consequences for brain structure and cognition than a decrease in adult neurogenesis. Indeed, juvenile neurogenesis was proposed to be indispensable in the formation of 25% of the total number of hippocampal granule neurons. Thereafter, in young adult and adult brains, the granule neuronal number remains stable and neurogenesis is only deemed to be necessary for cell replacement [70].

Previous research indicated that behavioural defects after *in utero* radiation exposure highly depend upon the evident morphological changes, such as brain volume decrease, ventricular enlargement and cortical thinning [71]. This is further evidenced in non-human primates, in which morphological effects induced by prenatal radiation exposure are thought to be responsible for neurological symptoms [72, 73]. Here, we observed both abnormalities in neurogenesis as well as morphological aberrations. Therefore, we cannot conclusively dissect the exact cause for inducing the cognitive defects. Moreover, as both alterations occur in different brain regions, an interplay and interconnectivity between distinct neuronal cell types may be responsible for the observed behavioural phenotype. For this reason, more behavioural tests addressing cortical versus hippocampal functions are recommended to segregate the functional outcome of an affected cortical and/or hippocampal development.

## Conclusions

In the present study, we used a multidisciplinary approach combining behavioural assessment, MRI, DTI, MRS, gene expression profiling and immunohistochemistry in order to reveal short- and long-term biological and molecular effects of radiation exposure during the early stages of mouse brain development. The observed long-term behavioural deficits and brain structure anomalies likely reflect the developmental defects initiated by radiation-induced DNA damage and subsequent apoptosis and inflammation. However, we could not provide a causal link between the early irradiation-induced apoptosis in the embryonic brain and adult behavioural and structural effects. Therefore, to fully correlate the adult phenotypic effects with prenatal apoptosis, experiments abrogating the early p53-dependent apoptotic response would be of use. In any case, we believe our study contributed to a better understanding of the timing and mechanisms responsible for radiation-induced neuronal non-cancer effects occurring several years after exposure. It also further highlights the importance and necessity of radioprotection during pregnancy, in particular during this critical time window of early neurogenesis.

## Electronic supplementary material

Additional file 1: Figure S1: Illustration of region selection for repeated quantification of MR imaging data in individual animals. (A) The cortical thickness was determined as the distance between the corpus callosum and the boundary of the brain in the axial view. To make the selection of the same location reproducible, we chose the slice where the lateral ventricles and the dorsal third ventricle apparently meet (left panel). Assessment of the cortical thickness up to 0.8 mm anterior or posterior to this location resulted in differences of the cortical thickness not larger than 2%–5% (right panel). (B) Illustration of the determination of the ventricle volumes using the ‘region-of-interest’ tool of ParaVision 5.1. Initial seeding points were set in the hyperintense regions of the ventricles. Lower and upper intensity thresholds were selected and the ‘volume growth’ algorithm was chosen for delineation of the ventricles. The selected volumes were then manually corrected. (C) Volumes for the determination of fractional anisotropy (FA) values were selected on 3D T2-weighted MR images. Those volumes were then transferred to the respective FA maps. Values were determined from regions in the corpus callosum (1), motor cortex (2), hippocampus (3) and thalamus (4). (TIFF 5 MB)

Additional file 2: Table S1: Primer sequences for quantitative reverse transcriptase PCR (qRT-PCR). Primers used for quantitative reverse transcriptase PCR qRT-PCR experiments included: *Cdkn1a*, *Ccl3*, *Eda2r* and *Tnfrsf10b. Gapdh* was used as a control. (XLSX 11 KB)

Additional file 3: Table S2: Quantification of metabolites based on localised MR spectroscopy. Single-voxel MR spectra from a cortical and subcortical (striatal) region were acquired for control and E11-irradiated animals at 40 weeks after birth. Quantification was performed relative to the non-suppressed water signal (expressed in μmol g^-1^). Data are presented as mean ± SEM. The number of animals used is indicated. (XLSX 12 KB)

Additional file 4: Figure S2: No change in cortical apoptosis and proliferation at 1 week post irradiation with 1.0 Gy. We analysed cleaved caspase-3 (CC3) (A) and PCNA (B) in brain sections from embryos irradiated with 1.0 Gy at E11, which showed no marked changes in expression when compared to the sham-irradiated animals. Data are presented as mean ± SEM. The number of animals used per test is indicated in the graphs (*N*). (TIFF 373 KB)

Additional file 5: Figure S3: No difference in hippocampal apoptosis and proliferation at 1 week after E11 irradiation. Cleaved caspase-3 (CC3) (A), PCNA (B) and Sox2 (C) immunoreactivity was quantified in sections of the embryonic hippocampus at 1 week post E11 irradiation with 1.0 Gy. This showed no alterations in expression between irradiated and sham-irradiated mice. Data are presented as mean ± SEM. The number of animals used per test is indicated in the graphs (*N*). (TIFF 464 KB)

## Abbreviations

CC: Corpus callosum
CC3: Cleaved caspase-3
ChIP: Chromatin immunoprecipitation
Chol: Choline
Cre: Creatine
DTI: Diffusion tensor imaging
E: Embryonic day
EPI: Echo planar imaging
FA: Fractional anisotropy
FOV: Field of view
GO: Gene Ontology
GPC: Glycerophosphorylcholine
H: Hippocampus
LV: Lateral ventricles
MRI: Magnetic resonance imaging
MRS: Magnetic resonance spectroscopy
MWM: Morris water maze
NAA: N-acetyl aspartate
PChol: Phosphorylcholine
PCr: Phosphocreatine
qRT-PCR: Quantitative reverse transcriptase PCR
RARE: Rapid acquisition with refocused echoes
SGZ: Subgranular zone
SMC: Sensory-motor cortex
T: Thalamus
TE: Echo time
TR: Repetition time.
