# Supplementary figures and images for: A multidisciplinary approach unravels early and persistent effects of X-ray exposure at the onset of prenatal neurogenesis
Source: J Neurodev Disord. 2015 Jan 9;7(1):3. doi: 10.1186/1866-1955-7-3 (PMC4448911; doi:10.1186/1866-1955-7-3)

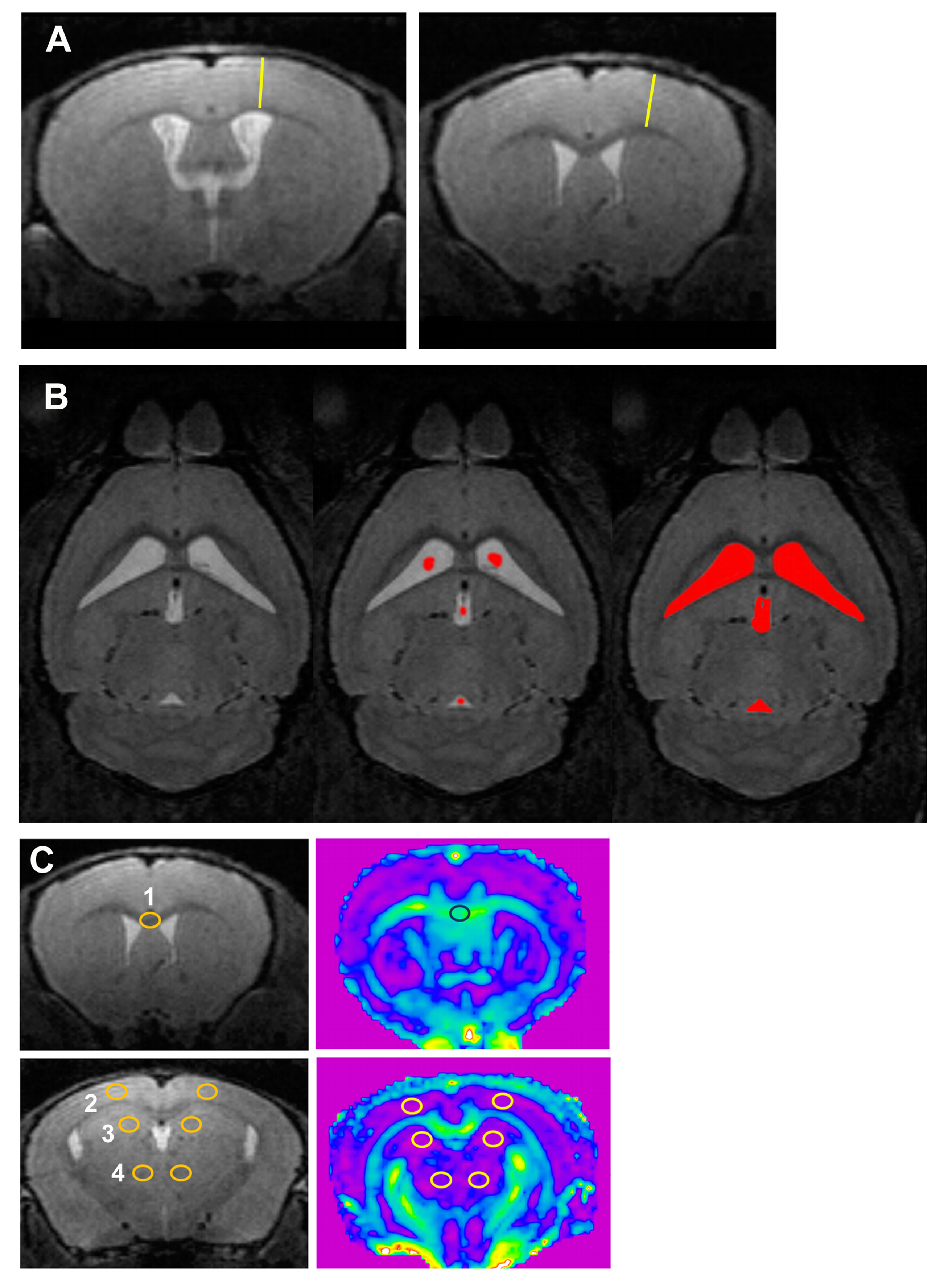

Supplement: Supplementary file 1 — Additional file 1: Figure S1: Illustration of region selection for repeated quantification of MR imaging data in individual animals. (A) The cortical thickness was determined as the distance between the corpus callosum and the boundary of the brain in the axial view. To make the selection of the same location reproducible, we chose the slice where the lateral ventricles and the dorsal third ventricle apparently meet (left panel). Assessment of the cortical thickness up to 0.8 mm anterior or posterior to this location resulted in differences of the cortical thickness not larger than 2%–5% (right panel). (B) Illustration of the determination of the ventricle volumes using the ‘region-of-interest’ tool of ParaVision 5.1. Initial seeding points were set in the hyperintense regions of the ventricles. Lower and upper intensity thresholds were selected and the ‘volume growth’ algorithm was chosen for delineation of the ventricles. The selected volumes were then manually corrected. (C) Volumes for the determination of fractional anisotropy (FA) values were selected on 3D T2-weighted MR images. Those volumes were then transferred to the respective FA maps. Values were determined from regions in the corpus callosum (1), motor cortex (2), hippocampus (3) and thalamus (4). (TIFF 5 MB) [file 11689_2014_110_MOESM1_ESM.tiff]

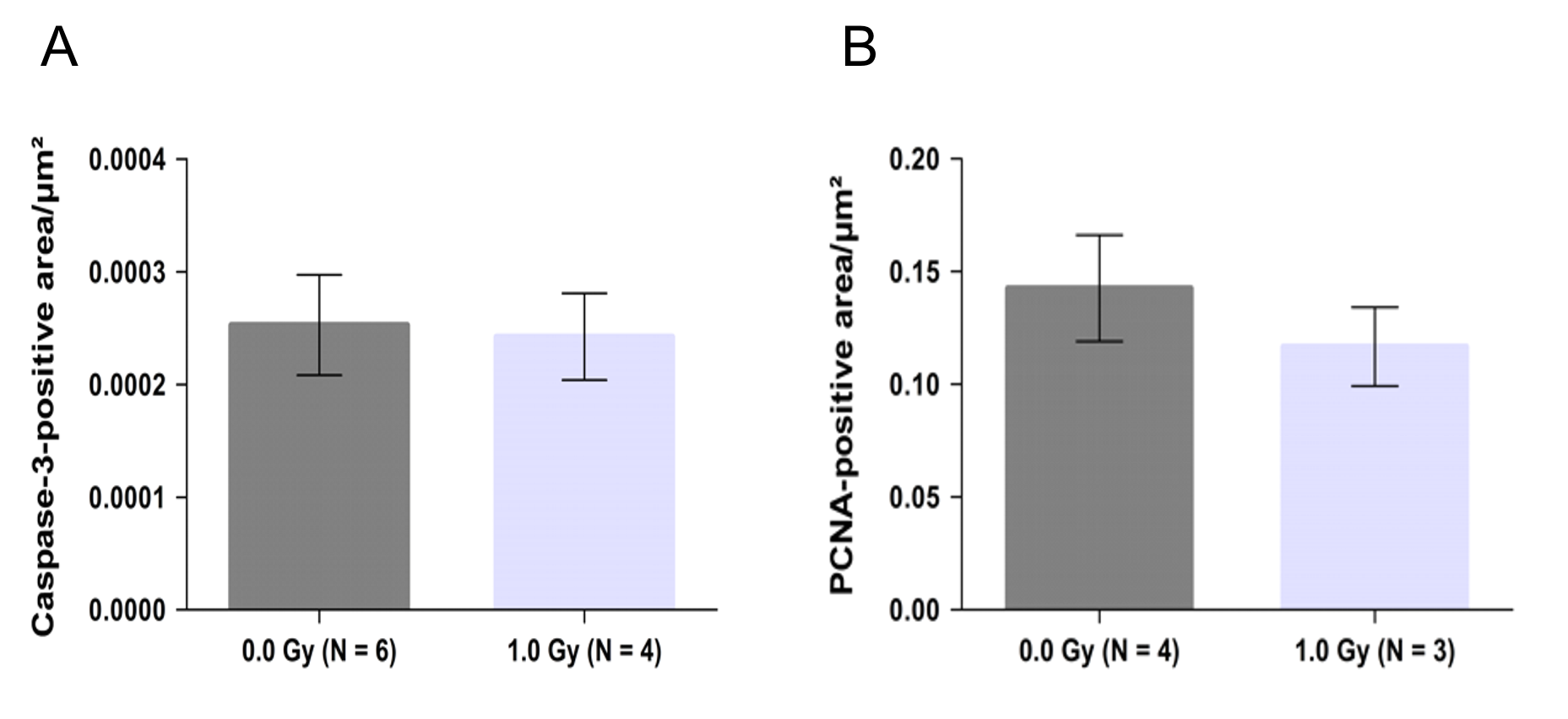

Supplement: Supplementary file 4 — Additional file 4: Figure S2: No change in cortical apoptosis and proliferation at 1 week post irradiation with 1.0 Gy. We analysed cleaved caspase-3 (CC3) (A) and PCNA (B) in brain sections from embryos irradiated with 1.0 Gy at E11, which showed no marked changes in expression when compared to the sham-irradiated animals. Data are presented as mean ± SEM. The number of animals used per test is indicated in the graphs (N). (TIFF 373 KB) [file 11689_2014_110_MOESM4_ESM.tiff]

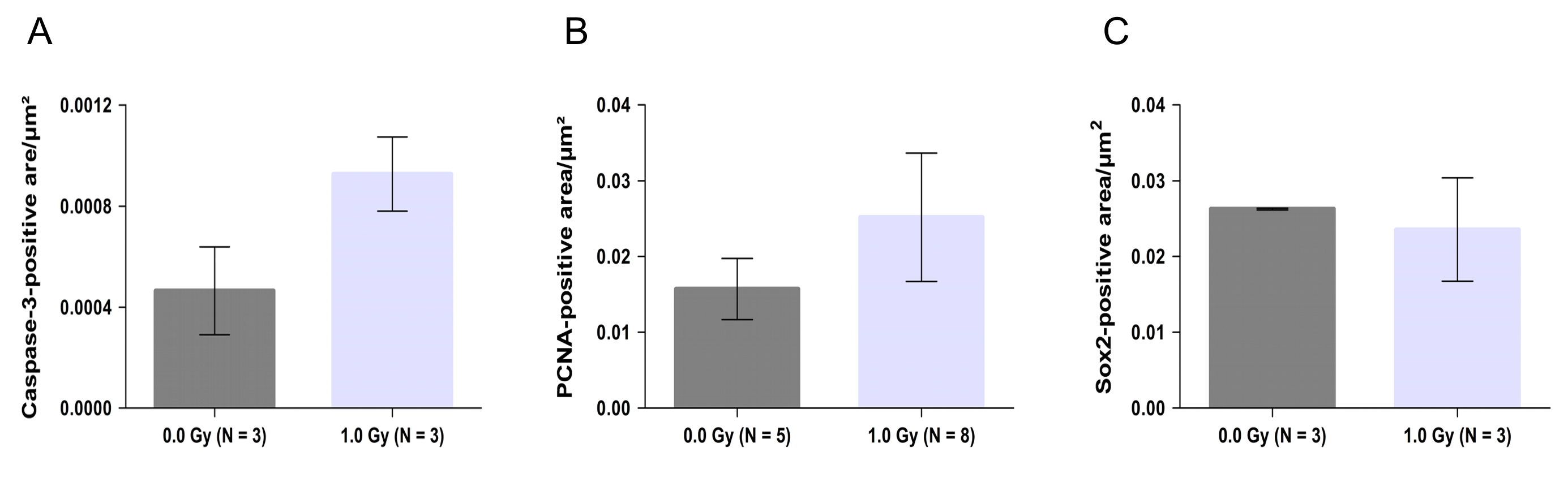

Supplement: Supplementary file 5 — Additional file 5: Figure S3: No difference in hippocampal apoptosis and proliferation at 1 week after E11 irradiation. Cleaved caspase-3 (CC3) (A), PCNA (B) and Sox2 (C) immunoreactivity was quantified in sections of the embryonic hippocampus at 1 week post E11 irradiation with 1.0 Gy. This showed no alterations in expression between irradiated and sham-irradiated mice. Data are presented as mean ± SEM. The number of animals used per test is indicated in the graphs (N). (TIFF 464 KB) [file 11689_2014_110_MOESM5_ESM.tiff]
